# Performance of two low-threshold population replacement gene drives in cage populations of the yellow fever mosquito, *Aedes aegypti*

**DOI:** 10.1371/journal.pgen.1011757

**Published:** 2025-06-26

**Authors:** Zachary J. Speth, David G. Rehard, Patricia J. Norton, Alexander W.E. Franz

**Affiliations:** Department of Veterinary Pathobiology, University of Missouri, Columbia, Missouri, United States of America; Peking University, CHINA

## Abstract

*Aedes aegypti* is the predominant vector for arboviruses including dengue, Zika, and chikungunya viruses, which infect over 100 million people annually. Mosquito population replacement in which arbovirus-susceptible mosquitoes in the field are replaced by laboratory-engineered refractory mosquitoes represents a novel genetic control measure to interrupt arboviral disease cycles. For this approach, the engineered mosquitoes need to harbor two genetic components: an antiviral effector construct which is linked to a gene drive (GD). We tested the performance of two single-locus CRISPR/Cas9 based GD for *Ae. aegypti* population replacement in small cage populations for up to 16 generations. Starting from a low release threshold of 1:9 GD bearing males to wild-type males, we observed two GD constructs in which Cas9 was expressed from two different germline promoters, *nanos* and *zpg*, to increase in frequency in all cage populations. By G16, an average of 72% and 82% of individuals from the *zpg*-GD and *nanos*-GD populations, respectively, harbored at least one GD copy with corresponding increases in allele frequencies. This indicated that the two single-locus, CRISPR/Cas9-based homing GD exhibited continuous super-Mendelian inheritance in populations of *Ae. aegypti*. Gene drive blocking indel (GDBI, a.k.a. “resistant alleles”) frequency was measured for each discrete generation in pooled samples from the six populations harboring GD. We found that populations with Cas9 expression under control of the *nanos*-promoter accumulated GDBI at more than twice the rate of those populations harboring the *zpg*-promoter driven GD. Based on preexisting data sets for homing and GDBI frequencies in addition to the cage trial observations, the relative contributions of sex-specific homing rates, maternal Cas9 deposition and potential fitness effects were modeled in MGDrivE for both GD, further explaining their divergent performance. Our study demonstrates the feasibility of low-threshold, single-locus CRISPR/Cas9 based GD for *Ae. aegypti* population replacement.

## Author summary

The mosquito *Aedes aegypti* is the major tropical vector for important human-pathogenic arboviruses. A novel genetic approach to suppress arbovirus transmission in the field includes population replacement with engineered virus-resistant mosquitoes. This approach requires the engineered mosquitoes to harbor two genetic components: 1) a potent antiviral effector that is 2) tightly linked to a gene drive (GD) system. Here we assess the performance of two single-locus, low-threshold GD in generational cage populations. Our data show that the two GD, which differ only by their germline specific promoter driving Cas9 expression, *nanos* or *zpg*, facilitated similar population invasion from low-threshold release conditions. However, in the cage populations, the *nanos*-GD generated more gene drive blocking indels (GDBI) over time than the *zpg*-GD. In continuous stochastic simulations, the *zpg*-GD resulted in a more robust population replacement than the *nanos*-GD. These results illustrate the importance of not just the homing frequency of a GD but also its GDBI formation rate, both of which strongly affect the stability of a GD in population replacement applications. Overall, our results suggest that autonomous, single-locus CRISPR/Cas9 based GD can be suitable vehicles for the sustained spread of antiviral effector transgenes in naïve populations of *Ae. aegypti*.

## Introduction

The yellow fever mosquito, *Aedes aegypti*, is the predominant vector for arboviruses including dengue (DENV), Zika (ZIKV), and chikungunya viruses (CHIKV), which infect over 100 million people annually [1]. *Ae. aegypti* is abundantly present throughout the tropical regions of the world including the southern United States [2]. *Ae. aegypti* was the principal vector responsible for a major ZIKV epidemic in Central-, South America and the Caribbean lasting from 2014-2017 [3]. *Ae. aegypti* is also the primary mosquito vector behind the largest dengue outbreak in South- and Central America, and South- and Southeast Asia on record, which started in 2023 [4–6]. Due to human activity and global warming, the distribution of *Ae. aegypti* populations in the western hemisphere is anticipated to expand in the following years within its tropical regions, and also northwards into more temperate areas [7]. These scenarios, as predicted, would likely be accompanied by a further spread of *Ae. aegypti* transmitted arboviruses. All this illustrates the need for efficient control strategies targeting *Ae. aegypti* and/or the arboviruses transmitted by this mosquito species. The efficacy of conventional mosquito vector control approaches, relying heavily on insecticide applications and mosquito habitat removal, is hampered by several factors including the presence of widespread insecticide resistance within *Ae. aegypti* populations, combined with the strong adaptation of this mosquito species to urban habitats [8–11]. Linking an anti-pathogen effector to a population replacement gene drive (GD) forms the genetic basis for an approach aiming at introducing a novel pathogen resistance trait into a targeted mosquito population [12–24].

Antiviral effector transgenes conferring resistance to DENV, ZIKV and CHIKV by utilizing engineered long inverted-repeat RNAs, microRNA arrays, single-chain antibodies, or hammerhead ribozymes have previously been demonstrated to suppress the replication and systemic infection of these arboviruses in *Ae. aegypti* [25–32]. Coupling an antiviral effector to a GD is an essential step to spread and maintain the effector transgene within a wild population of mosquitoes thereby preventing loss of the transgene between generations due to selection [33]. Previously, anti-*Plasmodium* effectors coupled to GD in *Anopheles* mosquitoes have been developed and shown in laboratory settings to effectively replace *Plasmodium* susceptible populations with parasite-refractory mosquitoes [22].

Gene drives are genetic elements which bias their own inheritance to super-Mendelian ratios [34,35]. CRISPR/Cas9 based GD bias inheritance through their homing endonuclease activity, causing the GD bearing allele to be copied to replace the wild-type allele on the homologous chromosome via homology-directed DNA repair (HDR) of Cas9 induced DNA double-strand breaks (DSB) [15–24,36]. While observed homing rates were often very high in *Anopheles* spp., in some cases resulting in greater than 95% transmission bias [18,19,22], lower homing rates have been observed for CRISPR/Cas9 based GD in *Ae. aegypti*, and most GD were designed for this species as split systems [20,37,38]. Split GD systems are confinable, but they would also require high release ratios of transgenic to wild-type insects, along with continuous releases over a sustained period, which may span several months in the field. In contrast, low-threshold, single-locus CRISPR/Cas9 GD can achieve high rates of population invasion from a single release of male mosquitoes as shown for *Anopheles* spp. [15,19,22]. Research on low-threshold single-locus GD in *Ae. aegypti* has thus far been limited to two studies, and the performance of such GD in cage trial populations of *Ae. aegypti* has not been tested prior to this work [21,39]. Low-threshold GD allow for the placement of both the GD components and antiviral effectors into a single, stable genomic locus, which is permissive for high levels of transgene expression [21,26]. Studies on GD within laboratory populations of mosquitoes have previously yielded new insights into the drive dynamics, including effects from parentally deposited Cas9 ribonucleoprotein complexes resulting in the formation of gene drive blocking indels (GDBI; a.k.a. “resistant alleles”) and their selection [38]. Small population studies additionally provide an empirical reference for GD models and help to reveal species and clade specific differences in GD behaviors.

Our study centers on the analysis of the performance of two low-threshold population replacement GD, *nanos*-GD and *zpg*-GD, including their measured invasiveness, homozygosity levels, and allelic resistance in small cage populations of *Ae. aegypti* mosquitoes over the course of 12–16 non-overlapping generations. Following data collection on the dynamics of the homing GD target locus, the cage trial outcomes are then compared to GD models, which are parameterized with sex-specific homing dynamics and maternal deposition effects.

## Results

### Generational inheritance of *nanos*-GD and *zpg*-GD in small cage populations of *Ae. aegypti*

**Fig 1:**
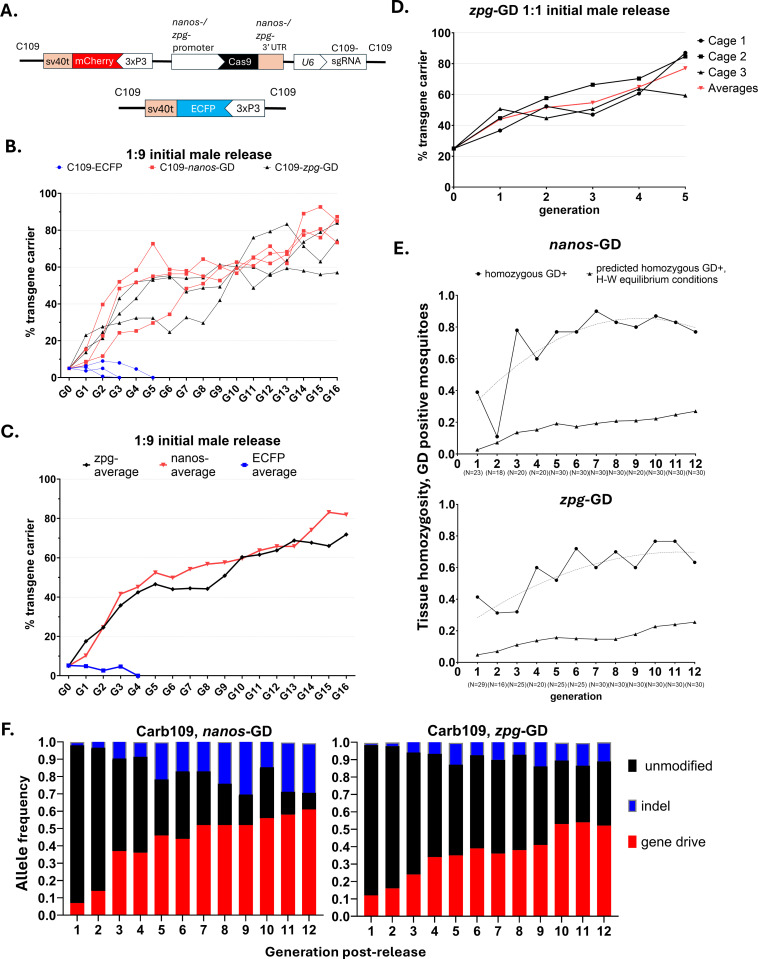
Single-locus CRISPR/Cas9 based GD exhibit continuous super-Mendelian inheritance bias in small cage populations of *Aedes aegypti.* **A.)** Schematic of the single-locus, CRISPR/Cas9 based GD transgenes, and the eye marker transgene (non-GD control) at the C109 locus [21]. **B.)** Heterozygous GD carrying *Ae. aegypti* males were released at a 1:9 ratio into populations of ‘wild-type’ (HWE) *Ae. aegypti* (Experiment A). Non-overlapping populations with discrete generations were maintained in triplicate for 16 generations following the introduction of either *nanos*-GD or *zpg*-GD. Populations consisted of 300 individuals throughout cage trials. During pupation, 150 individuals of both sexes were randomly picked and analyzed for eye marker expression and placed into cages to start the next generation. **C.)** Averaged results of 3 cage trial replicates for both GD and the control transgene shown in **B.)**. **D.)** The *zpg*-GD trial was repeated in triplicate with a 1:1 male release ratio (Experiment B). **E.)** GD carriers (Experiment A) were genotyped for 12 generations using an allele-specific PCR test. Both *nanos*-GD and *zpg*-GD exhibited super-Mendelian inheritance levels due to homing endonuclease activity, resulting in homozygote frequencies of GD carriers exceeding the expected frequency under Hardy-Weinberg equilibrium conditions in 11/12 generations for the *nanos*-GD and 12/12 generations for the *zpg*-GD. The *nanos*-GD exhibited greater rates of inheritance than the *zpg*-GD from G3-G10 (binomial test, method of small p-values). The dashed lines indicate homozygosity values for a smoothing function which was applied to calculate the GD allele frequencies. **F.)** Averaged allele frequencies from triplicate cage trial populations (Experiment A) harboring GD alleles, unmodified alleles, and gene drive blocking indels (GDBI, a.k.a. “resistant alleles”).

The two single-locus GD constructs each consisted of separate Cas9 and sgRNA expression cassettes and a fluorescent marker as cargo (Fig 1A). Cas9 was expressed from either *Ae. aegypti nanos* (AAEL012107) or *Ae. aegypti zero population growth* (*zpg; inexin-4*; AAEL006726) promoter sequences and 3’UTR. The GD constructs were site-specifically inserted on chromosome 3 in an intergenic locus (designated “Carb109” [26], or C109 [21]), which is permissive for stable antiviral effector gene expression. For reasons of simplicity, we will name our two GD transgenes from here on *nanos*-GD and *zpg*-GD.

Initially, we chose a low cage release ratio of GD bearing heterozygous males (1 GD male to 9 wild-type males at G0, Experiment A) to provide a sensitive test for GD performance over multiple generations. For both *nanos*-GD and *zpg*-GD, we observed over 16 consecutive non-overlapping generations of *Ae. aegypti* (strain: HWE) a substantial increase in the number of GD carriers throughout three cage trial replicates (Fig 1B). The average proportion of *nanos*-GD carrying mosquitoes increased from 10% of the male carriers at release (G0) to ~50% males and females carriers combined by G5 (Fig 1C). The *zpg*-GD exhibited a delayed relative increase in GD carriers, reaching ~50% invasion on average by G9. By G16, average invasion of the *zpg*-GD among the cage populations reached 72% (± 11.2%) while that of the *nanos*-GD amounted to 82% (± 6.1%) with males and females carrying at least one GD bearing allele (Fig 1B and 1C). Strikingly, a similarly introduced control transgene containing a fluorescent marker cassette at the C109 locus but lacking a GD was lost from all three replicate cage populations by G5. To test the hypothesis that C109 transgenic mosquitoes carry a baseline fitness deficit independent of the presence of the GD, we compared this result with a Wright-Fisher model of genetic drift with no selection (S1 Fig). Accounting for the effective population sizes of laboratory-adapted *Ae. aegypti* populations [40], we found that genetic drift was sufficient to cause the loss of the transgenic allele in absence of a GD. In Wright-Fisher model simulations with effective population sizes of 50–70 individuals, the allele, when introduced at a frequency of 2.5% was lost by G5 in 50–65% of 1000 total simulations (S1 Fig). However, this result does not rule out the possibility of an additional fitness cost associated with the transgene when inserted at the Carb109 (C109) locus [26].

We repeated the cage trial experiment for the *zpg*-GD with an increased GD-bearing male to wild-type male release ratio of 1:1 (Experiment B) (Fig 1D). The non-overlapping generation cage trials were then conducted under the same experimental conditions as described for Experiment A. Among the three cage replicate populations of Experiment B, the *zpg*-GD carrier frequency increased from 25% (males and females combined) at G0 to an average invasion of 77% (± 13%) by G5, an average increase of 52% from G0 to G5. By comparison, in Experiment A, the average *zpg*-GD carrier frequency at G5 was 44% (± 10%), an average increase of 39% from G0-G5.

### Homozygosity and GD allele frequencies for the *nanos*-GD and *zpg*-GD populations

Homozygosity for the *nanos*-GD and *zpg*-GD carriers was measured via an allele-specific PCR detection assay (Fig 1E). The allele frequencies of the single-locus GD were calculated for the six cage populations of Experiment A for 12 generations based on numerical recordings of GD carriers (using eye marker expression as a proxy) and their level of homozygosity as revealed by the PCR assay (Fig 1E and 1F). Whole-body mosquito samples carrying either *nanos*-GD or *zpg*-GD exhibited ratios of homozygosity for the transgene throughout the study period far exceeding the expectations for a population meeting the conditions for Hardy-Weinberg equilibrium (Fig 1E). This result was likely caused by the greater somatic zygosity of the GD carriers and does not necessarily indicate that the mating type of the tested individuals was homozygous. From G3 onwards to G12, we observed a higher proportion of the predominantly homozygous GD carriers among the *nanos*-GD cage trial populations in comparison to the *zpg*-GD populations. Between G3 and G10, 60–90% (79.7% on average) of adult *nanos*-GD carriers tested as homozygous. In comparison, from G3 onward to G10, samples from the *zpg*-GD individuals tested as 32–77% homozygous (60.9% on average), which was significantly less than the average *nanos*-GD carrier proportion (p ≤ 0.03, binomial test, with homozygosity ratios compared in increments of two generations over the interval; Fig 1E). The *nanos*-GD continued to generate populations with a greater proportion of GD bearing individuals testing as homozygous than the *zpg*-GD even in later cage trial generations, when mating opportunities between *nanos*-GD carriers and naive individuals were decreasing while GDBI accumulation was increasing throughout the populations. Taken together, these observations align with the greater overall homing rate of the *nanos*-GD at the C109 locus in comparison to the *zpg*-GD [21].

### Sex-ratios of GD carriers from the *nanos*-GD and *zpg*-GD cage populations

**Fig 2:**
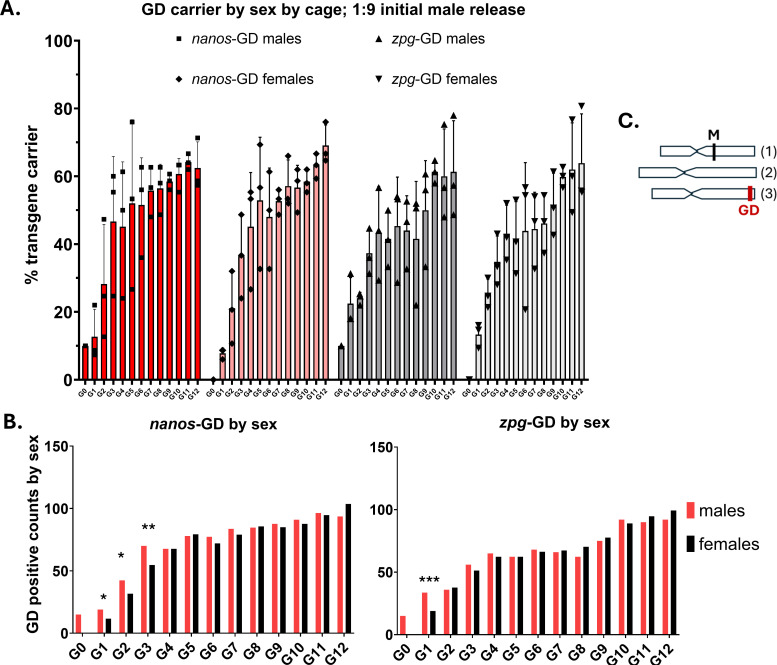
Sex ratios of *nanos*-GD and *zpg*-GD carriers from the cage trial populations. **A.)** Proportions of GD carriers by sex in triplicate cage trial populations harboring *nanos*-GD or *zpg*-GD (Experiment A). **B.)** Side-by-side comparisons of GD positive counts for male and female *Ae. aegypti* individuals in populations with 300 mosquitoes each. A significant sex bias for increased male inheritance of the GD was detected from G1 through G3 in the *nanos*-GD populations and G1 in the *zpg*-GD populations (Fisher’s exact test, * p ≤ 0.025, ** p ≤ .0025, *** p ≤ .00025). Bars represent the averaged values of three replicates for counts of male and female GD carriers out of 150 individuals per cage. **C.)** Schematic of *Ae. aegypti* chromosomes 1-3 with representative location of the male sex determining locus, marked M, on the first chromosome and the C109 GD target site on the third chromosome (marked GD).

In the first few generations following their initial release (Experiment A), we observed for the carriers of either GD a modest but significant sex bias towards male GD inheritance (Fig 2A and 2B). This male sex bias was observed between G1 and G3 among *nanos*-GD populations and among the G1 of the *zpg*-GD populations. There was no further evidence of sex-biased GD inheritance at later generations (Fig 2B and S1 Table). This pattern may have directly resulted from a male hereditary bias for GD homing or from a sex-dependent difference in viability associated with the GD transgene, which was lost during subsequent outcrosses to wild-type individuals from the cage populations. It is important to note that the GD were not located on the same chromosome containing the sex-determining locus of *Ae. aegypti,* which is located on chromosome 1 [41,42] (Fig 2C).

### Assessment of gene drive blocking indels (GDBI) in *nanos*-GD and *zpg*-GD cage populations

**Fig 3:**
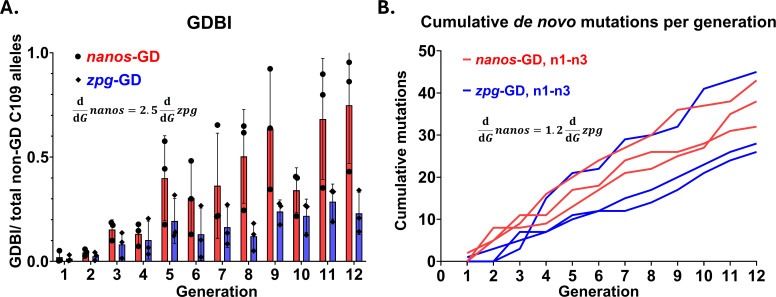
Cas9-driving promoter impacts gene drive blocking indel (GDBI) accrual caused by CRISPR/Cas9 GD activity in cage trial populations. **A.)** *nanos*-GD carrying populations exhibit a greater proportion of GDBI than *zpg*-GD carrying populations. Total DNA was extracted from 72 pooled samples of 100 larvae each, which were randomly collected for each cage trial replicate from generations 1-12 (Experiment A) of the cage trial. PCR amplicons (expected size 537 bp) spanning the GD target site were produced from the 72 samples of pooled genomic DNA. GDBI were measured by deep-sequencing the pooled PCR amplicons spanning the GD target insertion site. **B.)** Observed cumulative *de novo* mutations were counted for each cage trial population harboring *nanos*-GD or *zpg*-GD. Only those mutations, which were measured at frequencies greater than 0.5% of the reads per sample were considered in the analysis.

**Fig 4:**
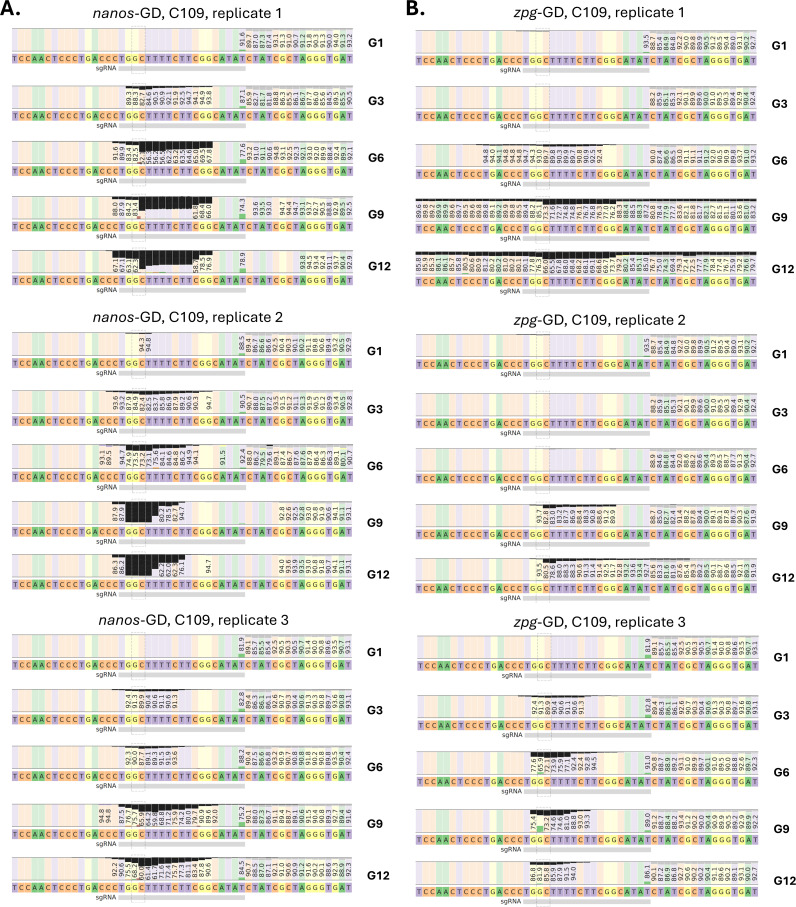
Presence of gene drive blocking indels (GDBI) at the sgRNA target site. Shown are representative generations of *Ae. aegypti* cage populations (Experiment A) harboring **A.)** *nanos*-GD and **B.)** *zpg*-GD. The fractions of aligned paired-end reads showing deletions at respective nucleotide positions are indicated by black bars.

To analyze GDBI formation following GD activity, DNA samples for 72 sets of paired-end sequencing reads were collected representing each cage trial population over 12 generations. Each DNA sample was prepared from pools of 100 larvae. Deep-sequencing results revealed striking differences in the accrual of GDBI in populations of Experiment A harboring the *nanos*-GD or *zpg*-GD (Figs 1F, 3A and 4). From G2 onward, *nanos*-GD populations exhibited a greater proportion of GDBI at every generation than *zpg*-GD populations. In addition, the proportions of GDBI as a share of non-GD alleles diverged over successive generations for the *nanos*-GD and *zpg*-GD populations. By G12, the *nanos*-GD populations had accumulated GDBI in most target alleles which did not carry the GD, with mutant alleles (GDBI) occupying >86% of non-GD alleles in two out of three *nanos*-GD populations (Figs 3A and 4). In contrast, *zpg*-GD populations accumulated GDBI at a lower rate, with mutant alleles occupying 23% on average (±8.2%) of the non-GD alleles at G12 (Fig 3A). The observed *de novo* indels for each population from the 72 sets of paired-end reads were further examined for unique mutations as well as patterns indicative of repair pathway choice (Fig 3B). Only those mutations which were occurring in more than 0.5% of reads, or >1 whole-body haplotype mass equivalent, were considered in the analysis to reduce the overcounting of mutations restricted to somatic tissues. This way, 120 unique *de novo* indels at the C109 locus were identified in reads from the six cage populations (S2 Table). The mutations primarily consisted of short deletions of <15 base pairs distributed 5’ of the protospacer adjacent motif (PAM), which is characteristic of non-homologous end joining (NHEJ) repair of Cas9 induced DSB [43]. This PAM-End Proximal Protected Repair (PEPPR) mutation pattern typically develops when the PAM-distal end of the DSB remains bound to Cas9 ribonucleoprotein for an extended period of time [44,45] (Fig 4 and S2 Table). Several mutations characteristic of PEPPR were repeatedly observed, including five short deletions at the target locus, which were found in all six of the cage trial populations (S2 Fig and S2 Table).

Thus, when comparing the two GD over 12 cage trial generations, the *nanos*-GD populations accrued GDBI at more than twice the rate of the *zpg*-GD populations (Fig 3A). However, when focusing on unique *de novo* mutation accumulations among the cage trial populations, both GD showed a much less divergent trajectory over time (Fig 3B). The identification of identical mutations among *nanos*-GD and *zpg*-GD populations (S2 Fig and S2 Table) suggests a certain bias in the DNA repair procedure that generates these recurrent GDBI types. Overall, the discrepancy in GDBI accrual between *nanos*-GD and *zpg*-GD populations could be caused by 1) greater activity of parentally deposited Cas9 in the *nanos*-GD populations; 2) selection for GD resistant mutations; or 3) an increased rate of introduced *de novo* mutations within the *nanos*-GD populations (Fig 4). The stronger overall mutation accumulation observed for *nanos*-GD may be the result of a greater parentally deposited Cas9 activity in the *nanos*-GD populations as previously observed ([21]; Table 1) or caused by selection.

**Table 1:** Gene drive model parameters.

Model	cF (rate of GD activity in the female germline)	cM (rate of GD activity in the male germline)	chF = chM (proportion of cF or cM resulting in gene drive)	crF = crM (proportion of cF or cM resulting in GDBI formation)	dF (rate of maternal deposition resulting in GDBI formation)	fL (GD- associated fitness cost, where (1-fL) is the fitness of individuals carrying a GD allele)
***nanos*-GD models parameters list**						
nanosM1	**0.361**	**0.419**	–	–	–	–
nanosM2	**0.361**	**0.419**	**0.93**	**0.07**	–	–
nanosM3	**0.361**	**0.419**	**0.93**	**0.07**	0.19	–
nanosM4	**0.361**	**0.419**	**0.93**	**0.07**	0.19	0.10
nanosM3s1	**0.361**	**0.419**	**0.93**	**0.07**	0.175	–
nanosM3s2	**0.361**	**0.419**	**0.93**	**0.07**	0.20	–
nanosM3s3	**0.361**	**0.419**	**0.93**	**0.07**	0.225	–
**nanosM3s4**	**0.361**	**0.419**	**0.93**	**0.07**	0.25	**–**
nanosM4s1	**0.361**	**0.419**	**0.93**	**0.07**	0.19	0.025
**nanosM4s2**	**0.361**	**0.419**	**0.93**	**0.07**	0.19	0.050
**nanosM4s3**	**0.361**	**0.419**	**0.93**	**0.07**	0.19	0.075
***zpg*-GD models parameters list**						
zpgM1	**0.200**	**0.119**	–	–	–	–
zpgM2	**0.200**	**0.119**	**0.97**	**0.03**	–	–
**zpgM3**	**0.200**	**0.119**	**0.97**	**0.03**	0.075	–
zpgM4	**0.200**	**0.119**	**0.97**	**0.03**	0.075	0.025
zpgM3s1	**0.200**	**0.119**	**0.97**	**0.03**	0.05	–
zpgM3s2	**0.200**	**0.119**	**0.97**	**0.03**	0.10	–
zpgM3s3	**0.200**	**0.119**	**0.97**	**0.03**	0.14	–

M1 model: with homing and no GDBI; M2 model: homing with GDBI introduced at low frequency in the germline; M3 model: homing with GDBI and maternal deposition; M4 model: M3 model with an additional fitness cost parameter. The complete GD model for M3 is shown in S1 Text; the fitness effect is applied by a separate masking function from the MGDrivE package. The homing rates are calculated from extensive single cross data [21], which is summarized in S3 Table. The underlined maternal deposition rates are initial estimates from two separate proxy tests for maternal deposition activity, which are summarized in S2 Text. The models which best fit to the observed GD dynamics in the cage trials are in **bold** and underlined.

### Modeling the performance of *nanos*-GD and *zpg*-GD

**Fig 5:**
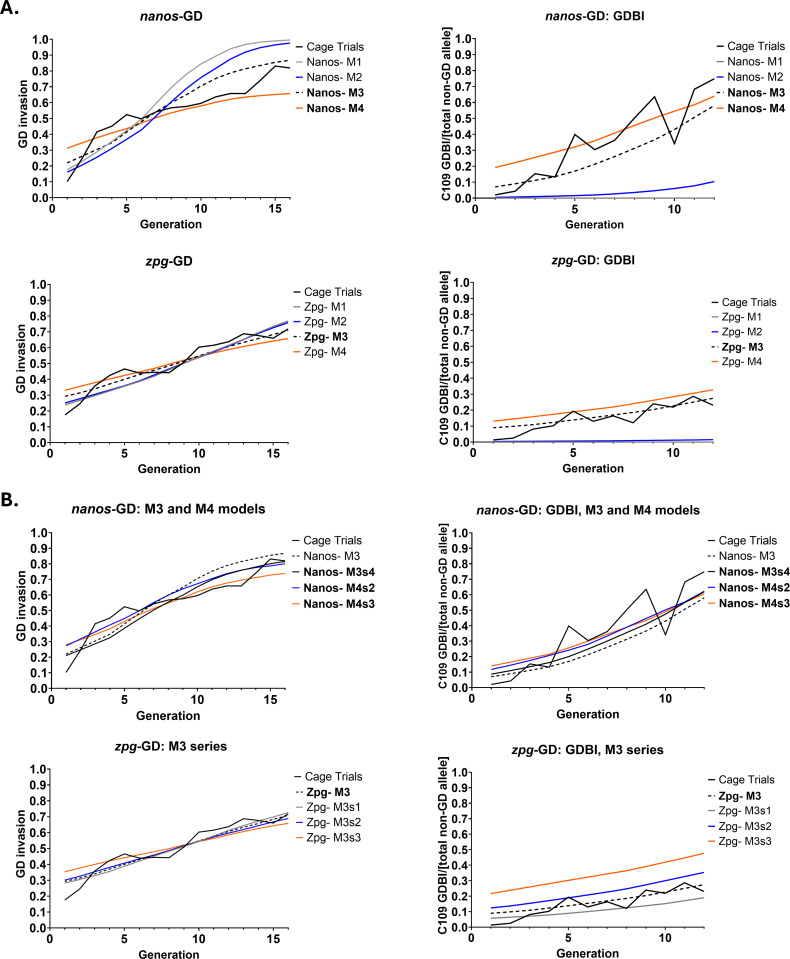
Observed versus modeled behaviors of the two single-locus, population replacement GD, *zpg*-GD and *nanos*-GD. **A**.) Simulation outcomes for fraction based, non-overlapping generation solutions were compared to cage trial results for up to 16 generations regarding invasion and for 12 generations regarding GDBI accrual. Both *nanos*-GD and *zpg*-GD, were modeled (using MGDrivE) with M1: GD homing only; M2: GD homing and GDBI formation from non-HDR events; M3: GD homing with GDBI formation from non-HDR events and additional maternal deposition effects leading to increased GDBI formation; and M4: the M3 model with an additional GD associated fitness cost. The model parameters are listed in Table 1. **B.)** Additional parametric sweeps (s1-s4) were applied to the M3 and M4 models to identify the best fitting model for either GD. Simulation outcomes are shown for the *nanos*-GD demonstrating the relationships between GD invasion and GDBI formation when accounting for maternal deposition effects with/without additional fitness cost. For the *zpg*-GD simulation, outcomes are shown demonstrating the relationships between GD invasion and GDBI formation when accounting for different maternal deposition rates in the absence of any fitness cost. The model alignments to the cage trial results are shown Table 2. In **bold** within the figure legends: type of simulation(s), which produced the best match with the observed average cage trial data.

According to GD invasion and GDBI accumulation observed in the cage trials, we modeled for both GD the relative contributions of sex-specific homing rates, maternal Cas9 deposition and potential fitness effects (S1 Text). Using the empirically measured values for the GD homing rates from Table 1 and S3 Table, we first established deterministic models for *nanos*-GD and *zpg*-GD (Fig 5). We tested four model variants (M1-M4) for each GD. In M1, only homing in the absence of any GDBI was considered. In M2, a low frequency of GDBI from Cas9 activity in the germline was assumed, while in M3, the complete model including GDBI produced by maternal Cas9 deposition was tested with *nanos*-GD and *zpg*-GD specific parameters. M4 includes M3 parameters plus an additional fitness cost. Models M1-M4 were then compared to the cage trial results in regard to invasion and GDBI formation (Fig 5A and Table 1). When assessing population invasion and GDBI accumulation for *nanos*-GD, M3 and M4 more closely matched the observed cage trial data than did M1 or M2 (Fig 5A). This was especially true for the performance of *zpg*-GD, which closely matched the M3 model in regard to GD invasion and GDBI formation when accounting for a maternal deposition rate of 7.5% (Fig 5A,5B and Table 1, Table 2). Adding lower (5%; zpgM3s1) or higher (10–14%; zpgM3s2, zpgM3s3) maternal deposition rates resulted in less-optimal curve fitting for this GD (Fig 5B). For *nanos*-GD, the situation was less unequivocal as M3 with 25% maternal deposition, M4 with19% maternal deposition plus 5% or 7.5% fitness cost (Table 1) all resulted in matches similar to the observed cage trail data (Figs 5B, S3 and Table 2).

**Table 2:** Gene drive model fit comparisons with cage trial data.

*nanos*-GD			*zpg*-GD		
Model	MASE (GD invasion)	MASE (GDBI formation)	Model	MASE (GD invasion)	MASE (GDBI formation)
nanosM1	2.924	2.561	zpgM1	1.087	2.844
nanosM2	2.504	2.312	zpgM2	1.066	2.681
nanosM3	1.488	0.863	**zpgM3**	**0.894**	**0.743**
nanosM4	1.339	0.816	zpgM4	1.164	1.329
nanosM3s1	1.615	0.903	zpgM3s1	0.894	0.992
nanosM3s2	1.418	0.844	zpgM3s2	0.954	1.437
nanosM3s3	1.259	0.791	zpgM3s3	1.256	3.549
**nanosM3s4**	**1.177**	**0.749**			
nanosM4s1	1.214	0.906			
**nanosM4s2**	**1.116**	**0.724**			
**nanosM4s3**	**1.019**	**0.747**			

The GD model parameters were scored by the mean absolute scaled error values (MASE). The models and parameters which best fit to either the *nanos-GD or zpg*-GD dynamics observed in the cage trials are indicated in **bold**.

**Fig 6:**
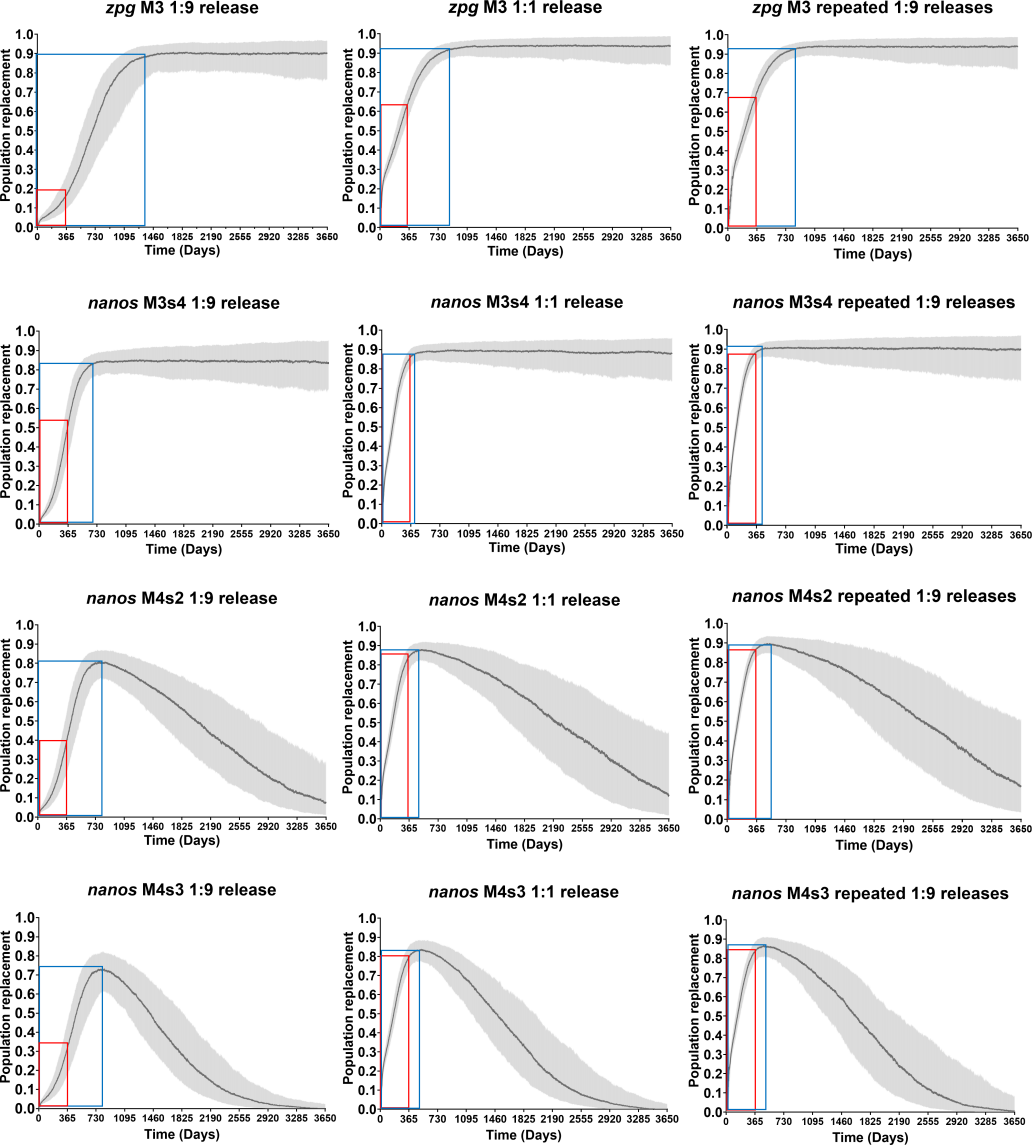
Performances of *nanos*-GD and *zpg*-GD in stochastic models considering continuous, overlapping mosquito generations and utilizing the optimal best fit parameters based on the deterministic models. The model assumptions for population structure and life history parameters are described in the methods and summarized in S5 Table. Three different release scenarios are shown, 1) single release of 1:9 GD-bearing males to HWE females; 2) single release of 1:1 GD-bearing males to HWE females; 3) repeated releases of 1:9 GD-bearing males to HWE females. The repeated releases consist of 10 releases interspersed by 4-day increments. The shaded gray area is the 90% confidence interval estimate from 500 simulations with an equilibrium population size of 500 *Ae. aegypti* (which is the median expected effective population size of *Ae. aegypti*). The darker lines show the median simulated population replacement at the specified timepoint. Frames in red: invasion (median values) at 365 days post-release. Frames in blue: timespan (days) until peak invasion (median values) has been reached.

We next compared how both GD would perform in a generalized field release scenario. To model this generalized scenario (i.e., no migration or seasonal variation in the life-history parameters or population growth rate, and an effective population size of 500 individuals [46]), we considered stochastic models with continuous, overlapping mosquito populations as targets for GD invasion. For the stochastic modeling of *zpg*-GD, we chose the parameterization according to the best fitting M3 model while for *nanos*-GD, we tested the parameters assigned for M3s4, M4s2, and M4s3 (Fig 5 and Tables 1 and 2). We simulated three different release scenarios for our GD-bearing mosquitoes: 1) single release of 1:9 GD-bearing to non-GD males; 2) single release of 1:1 GD-bearing to non-GD males; 3) repeated releases of 1:9 GD-bearing to non-GD males. For scenario 3), 10 releases are conducted at 4-day increments starting at day 0. For simplicity, we designate the releases “single 1:9”, “single 1:1”, and “repeated 1:9” from here on. It has been estimated that under ideal environmental conditions, *Ae. aegypti* can produce ~16 continuous generations per year in the field [47]. The MGDrivE model using our measured life history parameters would assume 15–20 continuous generations per year. Due to the lower homing rates, the *zpg*-GD requires about twice as many days (~730–1450 days post-release) than the *nanos*-GD without any assumed fitness cost (~380–750 days post-release) to approach a ~ 90% invasion in all three release scenarios (Fig 6). As a consequence of lower maternal deposition rates and greater fidelity, the *zpg*-GD reaches and maintains a slightly greater (median) peak invasion (90–93%) over time than the *nanos*-GD (85–90%). The scenario “single 1:9” generates the weakest population invasion rates per time period and the lowest level of fidelity for both *zpg*-GD and *nanos*-GD (Fig 6). The scenarios “single 1:1” and “repeated 1:9” generate ~63–68% (median) invasion within 1-year post-release for *zpg*-GD compared to ~90% (median) invasion for *nanos*-GD in the absence of any fitness cost (M3s4). If the *nanos*-GD were affected by just minor fitness costs (assumed to be 5% for *nanos*-GD M4s2 and 7.5% for M4s3), which cannot be ruled out based on the cage trial performance data, the GD would become highly unstable at ~1.5-2 years post-release. Ten years post-release, less than 20% of the individuals in the target population on average would be still GD carriers in presence of a 5% fitness cost. When assuming a 7.5% fitness cost, there would be no more GD-bearing individuals on average in the targeted population after 9 years post-release. These results demonstrate that the *zpg*-GD has a lower homing rate and requires a longer time span to reach a nearly complete population invasion, while overall being more robust than *nanos*-GD. The latter has higher homing rates, which also lead to higher GDBI accumulation levels over time. As shown for *nanos*-GD, the addition of relatively low fitness costs (5-7.5%) would destabilize the GD at <2 years following its introduction into a target population.

## Discussion

CRISPR/Cas9 based GD applications in *Ae. aegypti* have primarily focused on split designs in which the GD construct is divided over two different genomic loci, to test the effects of promoter choice on GD homing rates, GDBI formation and fitness cost [20,37,38]. Split GD function has been incrementally improved through the selection of germline specific promoters and multiplexing of sgRNAs [37]. However, so far, only two previous studies report single-locus, site-specifically integrated CRISPR/Cas9 GD in *Ae. aegypti* [21,39]. Our work here extends upon the study by Reid et al. [21] by adding a multi-generational cage trial experiment to monitor invasion, GDBI formation, and homozygosity levels for two single-locus GD, *nanos*-GD and *zpg*-GD, in which Cas9 expression was controlled by two different germline promoters. The measured time series data from the cage trials for invasion and GDBI formation was then compared to GD models to further explore how promoter dependent GD activity would affect overall GD performance in potential population replacement applications. In our cage trials, the *nanos*-GD and *zpg*-GD reached an average invasion of 82% and 72%, respectively, at G16 under a 1:9 release scenario of GD bearing males to wild-type males, and 77% on average under a 1:1 release scenario by G5 as observed for *zpg*-GD. These results clearly show that in *Ae. aegypti*, low-threshold CRISPR/Cas9 based homing GD facilitate transgenic allele invasion to a super-Mendelian equilibrium thereby overcoming allelic loss due to genetic drift. Indeed, genetic drift was a likely cause for the loss of the marker transgene bearing (at the C109 locus) individuals lacking a GD from the cage populations after five generations, when introduced at a 1:9 ratio of heterozygous transgenic males to wild-type males (S1 Fig).

Previous studies in *Anopheles* spp. and *Ae. aegypti* have investigated the effects of Cas9 promoter choice on GD activity [20,21,37–39,48]. Different promoter-dependent spatio-temporal Cas9 expression patterns affected homing rates, maternal deposition leading to GDBI formation, as well as GD activity in somatic tissues, which could lead to fitness reductions [49–51]. For *Ae. aegypti*, it should be noted that except for two of the studies [21,39], the GD transgenes containing different promoters were integrated into different chromosomal loci due to transposon mediated transformation resulting in a quasi-random choice of the transgene integration site [38,52]. This is an important aspect as position effects in *Ae. aegypti* are known to contribute to transgene instability [26,53] and may result in aberrant GD expression and function [38]. The C109 target locus in *Ae. aegypti* is located downstream of the distal-most protein encoding sequence (AAEL010318) on chromosome 3 [21] and has previously been shown to be a genetically stable integration site for antiviral effectors [26]. The *nanos*-GD and *zpg*-GD, when integrated at the same locus in *Ae. aegypti* exhibited different behaviors, particularly with respect to GDBI accumulation within populations (Fig 3). In *Ae. aegypti* females, *nanos*-transcripts are produced by the follicular nurse cells during oogenesis and deposited into the early embryo [54–56]. In *Anopheles* spp., *zpg* (=*innexin4*) transcripts have been shown to be expressed in both the male and female germlines to facilitate gonad development [48,57]. Earlier, it was shown that both *nanos* and *zpg* transcripts were detectable in the early embryo of *Ae. aegypti* for up to 48 h post-oviposition [58]. CRISPR/Cas9 GD inheritance and long-term fidelity in a host organism is dependent on the type of DNA repair mechanism utilized. HDR maintains a high level of GD fidelity due to its precise copy-paste mechanism whereas NHEJ leads to GDBI formation and accumulation. Spatio-temporal patterns of promoter driven Cas9 expression in zygotes, gametes, during early embryogenesis, and/or in the germline progenitor cells [49,51] dictate which DNA repair mechanism is utilized. In *Drosophila* and *An. gambiae,* Cas9 expression from the *nanos*-promoter led to a build-up of GDBI through maternally deposited Cas9 causing unfavorable NHEJ-mediated DSB repair [59,60]. The same mechanism, i.e., Cas9 ribonucleoprotein deposition from the follicular nurse cells, likely contributed to the higher rates of GDBI formation observed in our *Ae. aegypti nanos*-GD cage populations. Furthermore, in *Drosophila* harboring split GD or a single-locus GD, Cas9 deposition into individuals in which a genomic Cas9 source was absent has been shown to lead to homing of the sgRNA gene [61–63]. The deposited Cas9-sgRNA ribonucleoprotein complexes can be sufficiently stable to persist in an active state in the following generation [63]. This way, deposited Cas9 ribonucleoprotein can result in copy events of GDBI in heterozygotes carrying both GDBI containing and wild-type (“indel-free”) alleles. Indeed, when both GD of our study were modeled based on promoter-specific homing rates, differences in the maternal deposition rates were sufficient to account for most of the observed increases in GDBI formation in the *nanos*-GD populations (Fig 5 and Table 2).

Inheritance of *nanos*-GD and *zpg*-GD generated high ratios of homozygous individuals throughout the 12 test generations, which were significantly greater than the expected values under Hardy-Weinberg equilibrium assumptions (Fig 1E). It is likely that many of the individuals testing as homozygous in our genotyping PCR assay would in fact have mixed alleles in their germline, which our PCR assay would be unable to resolve. However, there was an interesting discrepancy between the two GD, with the *nanos*-GD showing greater homing rates and generating more homozygotes among the cage trial populations than the *zpg*-GD. This pattern is consistent with homing occurring in the early embryo following deposition of Cas9 ribonucleoprotein, which would generate predominantly homozygous somatic tissues although this is not the hereditary mechanism of the GD. In addition, homozygosity increased from G1 to G4, further indicating that maternal deposition effects may have enhanced the activity of the *nanos*-GD. Others have observed lethal mosaicism due to activity from maternally deposited Cas9-ribonucleoprotein [63] when acting upon essential host genes. The two GD of this study, which target an intergenic locus on chromosome 3 are likely not producing any lethal mosaicism. However, it can be speculated that both GD would have the potential to produce lethal mosaicism if targeting an essential gene in *Ae. aegypti*.

Observations on GDBI accrual within the cage trail populations indicated the presence of GD activity from maternally deposited Cas9 ribonucleoprotein. This was particularly evident for the *nanos*-GD. Although both GD showed similar levels of unique indel mutations within the populations, *nanos*-GD populations accumulated overall GDBI at greater rates. The *nanos*-GD matched those models in which maternal deposition rates were in >2.5-fold excess when compared to those models that best matched the *zpg*-GD performance (Fig 5 and Tables 2 and S4). This suggests that large quantities of GDBI among later generations of the *nanos*-GD bearing populations were generated via copying of the GDBI containing allele within the germline, from offspring receiving maternally deposited Cas9 ribonucleoprotein but lacking the actual GD allele. We also considered that selection for GDBI, arising from GD associated fitness cost would lead to a similar pattern of GDBI accrual, at least for *nanos*-GD. For this GD, moderate fitness deficits of 5-7.5% could not be ruled out, which would have strong destabilizing effects on the GD as shown in our stochastic models (Fig 6).

To better understand how both GD would perform in a field trial scenario given the respective combinations of homing rates, maternal deposition, and GD-associated fitness cost, we modeled the GD performances following various release conditions in continuous and mixed populations with overlapping generations and stochastic effects. We conducted the modeling primarily to compare both GD, and for simplicity we considered a generalized scenario without accounting for seasonality, regional differences in mosquito population growth rates, or migration between neighboring populations. We found that the *zpg*-GD, despite its lower homing rate, looked more favorable for longer-term population replacement, producing a more favorable balance between transgene invasion and GDBI accumulation rates. As pointed out above, the *nanos*-GD might become unstable as a result of self-imposed fitness effects, which has been shown before [21] and also for GD in other species [50]. However, although the *zpg*-GD looks more promising as a vehicle for longer-term introduction of a hypothetical gene of-interest, such as an antiviral effector, the GD had a relatively low replacement rate of just 11.8% (of the total population) per 100 days between days 100 and 500 following a “single 1:1” release. In comparison, in the simplified population structure scenario, the *nanos*-GD replaced 15.6% of the total population per 100 days between days 100 and 400 following a “single 1:1” release (M3s4 model, no fitness cost considered). Although both single-locus GD were designed for a low-threshold release scenario, they would require either serial releases, or single releases of large numbers of male GD carriers to achieve invasion of 66% for the *zpg*-GD or 86% for the *nanos*-GD within 1 year post-release, without factoring in any GD associated fitness cost (Fig 6).

Altogether, this work demonstrates that current single-locus *Ae. aegypti* GD, even with low homing rates and relatively high maternal deposition may be functional for long-term population replacement applications, for instance as vehicles for antiviral effectors. With regards to more rapid and complete replacement, the GD stand to improve from Cas9 and sgRNA regulatory element choices [64]. Furthermore, as shown previously and in this work, the simple single-locus GD design is sensitive to GD or transgene associated fitness costs [21,50]. Future efforts to improve *Ae. aegypti* GD may therefore benefit from a focus on design principles, which address fitness imbalances between the GD and GDBI. Alternative population replacement GD strategies for *Ae. aegypti*, which mitigate the effects of selection against GD due to associated fitness costs include Cleave and Rescue (ClvR), or Home and Rescue (HomeR) [17,18,23,65]. HomeR and ClvR are designed to decrease the fitness of GDBI by simultaneously increasing the relative fitness of the GD transgene via targeting/disrupting and eventually restoring an essential gene function. HomeR or ClvR targeting haplo-sufficient genes produce a fitness inequality, which causes a selection bias in favor of the GD [18,23]. Thus, while our single-locus *zpg*-GD and *nanos*-GD have clearly demonstrated the effective replacement of small *Ae. aegypti* populations in the short term, incorporating strategies for *Ae. aegypti* GD, which address fitness imbalances should further improve GD robustness and decrease any risk from unanticipated fitness constraints.

## Materials and methods

### Cage trials

Colonies of the *Ae. aegypti* Higg’s White Eye (HWE) strain and the outcrossed transgenic lines AeaNosC109GD [AF-35], AeaZpgC109GD [AF-46], and AeaCFPC109 [AF-18] (here referred to as *nanos*-GD, *zpg*-GD and C109-ECFP, respectively; [21]) are the sources of the mosquitoes used in this study. For the discrete, non-overlapping cage trials, founder populations of heterozygous males were established separately for each cage and transgenic line by outcrossing 20 transgenic males to 100 HWE females. This outcrossing step was conducted to remove those indels, which may have accumulated independently during routine maintenance of each GD line. G0 populations of each replicate cage trial in Experiment A were then established at a 1:9 GD male to wild-type (HWE) male release ratio by selecting 15 male transgenic pupae, 135 male HWE pupae, and 150 female HWE pupae, and introducing them together in 1-cubic foot cages (3 replicates were prepared). For Experiment B (1:1 GD male to wild-type male release ratio), 75 male transgenic pupae, 75 male HWE pupae, and 150 female HWE pupae were introduced together in each replicate cage. Male pupae of the transgenic lines and HWE were reared at the same time and collected on the same day to prevent any mating advantage when emerging into adults. Throughout our experiments, male pupae were collected earlier than female pupae due to a difference in the average developmental time to reach the pupal stage.

Rearing and counting of individuals for cage trials was performed as described in the following: mosquito larvae were reared in a controlled environment (28^o^ C, 80% humidity, 12 h dark, 12 h light cycle) and fed on tropical fish food (Cichlid Flakes; Tetra GmBH, Melle, Germany). At pupation, individuals were screened for mCherry or ECFP (non-GD control) eye marker expression. From G1 through G16 of the cage trials (Experiment A) and G1 through G5 in Experiment B, 150 male and 150 female pupae were randomly picked from each respective cohort. The number of individuals showing fluorescent eye marker expression was counted for each sex, and pupae were placed in emergence cups within 1 cubic-foot cages. Adult mosquitoes were supplied with raisins and water *ad libitum*. Eight days following pupation, adult females were fed on defibrinated sheep blood (Colorado Serum Company, Denver, CO, USA) using custom-made glass feeders. For the cage trials, females were monitored for blood-feeding status and re-fed the next day if fewer than 80% of females had taken a blood meal. Egg papers were placed in cages from days 2–6 post-blood feeding. After removal, eggs were allowed to mature for >4 days under drying conditions prior to hatching under vacuum. Mosquitoes were reared by hatching eggs from the previous generation and randomly selecting 500 larvae for rearing at ~125 individuals per tray (at a density of ~250 larvae per liter).

### Genotyping assay

Genotyping was performed on mosquitoes of Experiment A from G1 through G12 with an allele specific PCR test on randomly sampled male GD carriers from each replicate. Males were screened for the presence of the mCherry eye marker, and ~10 eye marker positive males per cage were frozen at -20^o^ C. Total DNA was extracted from individual carcasses using the Quick DNA Miniprep Plus kit (Zymo Research, Irvine, CA, USA) and eluted into Buffer EB (Qiagen, Germantown, MD, USA). In the PCR assay, 3 oligo-primers were used, which produced two differently sized amplicons for the native C109 locus (592 bp) and the same locus when harboring the GD insertion (336 bp). Results were combined for the 3 replicates of the *nanos*-GD and *zpg*-GD populations to estimate the allele frequencies over time. PCR conditions were as follows: template genomic DNA was added to GoTaq green master mix (Promega, Madison, WI, USA) with 6.7 picomoles primer F1_gty, 3.3 picomoles primer F2_gty, and 10 picomoles primer R_gty. Thermocycling conditions were the following: 95^o^ C for 1 minute, followed by 36 cycles of 95^o^ C for 30 sec, 55^o^ C for 20 sec, 72^o^ C for 40 sec; with a final extension period of 2 minutes at 72^o^ C. PCR products and 100 bp plus ladder (GoldBio, St. Louis, MO, USA) were loaded into 1.7% agarose gels and electrophoresed at 90V for 1 hour. Gels were stained with ethidium bromide and imaged on a FluorChem Q gel imaging system (Cell Biosciences, Santa Clara, CA, USA). The 3 PCR primers for genotyping were:

F1_gty (C109, no GD insertion): TCGCACCTAATCAGACAGTCG;

F2_gty (C109 with GD insertion): GAGCAGAGGCAAGAGTAGTG;

R_gty (outside the C109 GD insertion locus): CCTGCCTTCATTAAGCTCTTTG.

For each generation and GD, the Hardy-Weinberg equilibrium frequency was calculated from the combined observations from triplicate cage trials including those individuals that were homozygous for the recessive allele (i.e., no fluorescent marker allele). The set of equations p + q = 1 and 1 = q^2^ + 2pq + p^2^ was then solved for the expected frequencies of GD (dominant) and non-GD (recessive) alleles, and the associated genotype frequencies, under Hardy-Weinberg equilibrium conditions. To calculate the averaged allele frequencies in cage trial populations harboring the *nanos*-GD and *zpg*-GD (Fig 1F), a second order polynomial function was fitted to the homozygosity assessments based on the 3-primer PCR assays and averaged for 2 generations at a time in order to reduce noise from small sample sizes (S4 Fig). Binomial tests to assess differences in genotype distribution were calculated using the binomial test function in GraphPad Prism v10.4. To increase the power of the test, values were compared in two generation increments (that is, G1/2, G3/4, etc.) for both comparisons between cage trial homozygosity and Hardy-Weinberg equilibrium conditions, and between the two GD. In cases of uneven sample numbers, the lesser number of observations was used by normalizing the results of the experiment with the greater value of observations. The p-values shown are from the two-sided test statistic (binomial test, method of small p-values).

### Quantitation of gene drive blocking indels (GDBI)

We measured allele frequencies around the sgRNA target locus by paired-end Illumina deep-sequencing of target PCR amplicons using total DNA obtained from pools of 100 randomly selected second instar larvae. These originated from the ~ 500 individual larvae reared during each generation (until G12; Experiment A) for each cage. As a negative control, 100 larvae pools were collected from the parent HWE line. Total DNA was extracted from larval pools using the Quick DNA Miniprep Plus kit (Zymo Research) and eluted into Buffer EB (Qiagen). The primers utilized to produce the target amplicons bracketed the sgRNA target site by >250 bp in both 5’ and 3’ directions. The primers were predicted to produce a 537 bp amplicon around the C109 locus, when indels were absent. Amplicons were produced using Q5 High Fidelity PCR polymerase (NEB #M0491; New England Biosciences, Ipswich, MA, USA) on a Bio-Rad C1000 Touch Thermocycler using a 2-step PCR protocol: 10 cycles with 10 seconds of DNA denaturation at 98^o^ C, 20 seconds primer annealing at 56^o^ C and 30 seconds extension at 72^o^ C; followed by 20 cycles with the annealing temperature increased to 69^o^ C. The PCR products were analyzed on 1.5% agarose gels; bands were confirmed via ethidium bromide staining and the gel portion showing band signals between 450 bp and 750 bp in size were excised. The DNA was purified and concentrated from agarose gels using the Nucleospin Gel and PCR Clean-up kit (Takara Bio, San Jose, CA, USA) and samples were normalized to 1ng/μl using a Qubit fluorometer (Thermo Fisher Scientific, Waltham, MA, USA). Second round PCR assays using unique dual index sequencing primers and sequencing of resulting PCR amplicons were performed at the University of Missouri Genomics Technology Core. There, amplicons were sequenced on an Illumina MiSeq platform, producing 300 bp paired-end reads (PE300).

The resulting paired-end reads were trimmed using Trimmomatic [66]. Indel analysis was performed on the trimmed reads using Crispresso2 [67,68], with the minimum average read quality set to 10 and GGATAGCCGAAGAAAAGCCA defined as the C109 target specific crRNA sequence. Substitutions were ignored as part of the indel analysis, as multiple single-nucleotide polymorphisms (SNPs) were detected in controls 3’ of the sgRNA target site. GDBI were quantified for each pool as the percentage of reads with insertions or deletions within the sgRNA target site. For the quantitation of *de novo* indels, only mutations which were observed in >0.5% of all paired-end read counts in a sample were included. This threshold was determined from the sample size of 100 individuals (that is, 200 haplotypes per sample). Only the first observation of a unique mutation exceeding this threshold was counted as a *de novo* indel for each separate population. Over subsequent generations from the cage trials, the number of haplotypes per sample decreased as a result of GD replacement of wild-type alleles (since there was no selection of larvae based on eye marker expression ). Observed unique reads may also have resulted from somatic mutations, but these were less likely to reach the counting threshold.

The 1st round PCR primers for Illumina MiSeq, quantitation of indels were: F1_C109amplicon: ACACTCTTTCCCTACACGACGCTCTTCCGATCTATGGAAACAAAACACAAGGCATACA and R1_C109amplicon: GTGACTGGAGTTCAGACGTGTGCTCTTCCGATCTTTATCCGACGAAAATGTGTTCACTG (the gene target-specific nucleotide sequences are underlined). The Illumina MiSeq platform produced on average 177,000 paired-end reads per sample. The minimum read coverage for any sample was 13,481 paired-end reads, equivalent to 67x read coverage of the target amplicon. In total, amplicons from 7200 individual mosquitoes originating from 72 separate pooled samples, which were collected from the six GD cage populations were included in the analysis.

### Modeling of GD performance

Modeling studies were performed using the MGDrivE and MGDrivE2 packages with R studio for a population replacement homing endonuclease-like GD with sex-specific homing rates, non-negligible GDBI formation rates, and maternal deposition resulting in GDBI [21,69]. The model was updated from a previous version (CRISPR2MF.R) to correctly compute the expected offspring distributions following state transition (S1 Text). The sex-specific homing rates used in all models for either GD were calculated from a large collection of single-cross data (summarized in S3 Table) [21]. For all models tested in this study, the homing rates were considered fixed (Table 1).

The M1 model was reduced by specifying only the homing parameters (cF and cM parameters, and with chF = chM = 1) and with all other parameters equal to 0. For the M2 models, GDBI were added at a low frequency in the germline following GD induced DSB (crF and crM parameters, with crF = crM > 0, and with (cr + ch)=1). The M3 model included all of the parameters from M2, in addition to maternal deposition (specified by dF > 0). For this study, the maternal deposition was modeled only with dhF = 0 and drF = 1, assuming that it resulted in GDBI accumulation while not contributing to GD homing. For the M3 models, empirically measured GD specific maternal deposition rates (S2 Text) were applied as initial estimates, around which parametric sweeps were conducted to identify the best fitting parameters. The MGDrivE simulations were implemented as mixed population models (i.e., at any one time in the model there were mosquitoes in different life stages which included egg, larva, pupa and adult stages [69]) for both the fraction based, non-overlapping population simulations and stochastic, continuous population simulations. Only the results for the adult mosquito populations were reported. For the GD model including fitness cost, individual GDBI arising from NHEJ repair of CRISPR/Cas9 catalyzed DSB at the targeted intergenic locus were assumed to all have the same fitness. In the model (S1 Text), six different genotypes and 30 different mating combinations resulting from the three allele types of GD (“H”), wild-type (“W”), and resistant alleles/GDBI (“R”) were possible. The transition matrices (state transition matrices for the deterministic models, or stochastic matrices in the stochastic continuous population simulations) were defined for the specific GD behavior for each of the 30 mating combinations (S1 Text). Fitness cost was implemented using a separate function in the MGDrivE package, cubeModifiers [69,70]. Fitness cost reductions for the M4 models as shown in this study were implemented in the pupal stage by setting the successful pupation rates (xiF and xiM parameters) to less than 1 for each of the three genotypes harboring a GD allele, according to the fL value (reported in Table 1). The fitness cost was implemented for the pupation rate parameter based on reduced pupation rates of progeny resulting from *nanos-*GD outcrosses as had been observed earlier [21,70]. The complete GD parameters used for the modeling shown in the plots are listed in Table 1. The life history parameters used for the simulations are listed in S5 Table.

For the stochastic model, 500 simulations were run in stochastic mode in MGDrivE for each of the specified GD models and parameter sets (Table 1). For the generalized field release scenario tested with the stochastic modeling, we considered only a simplified population structure, with no migration, and generalized life history parameters (S5 Table) with no time dependent (seasonal) variation. The genotype data from the multiple simulations of each run was then compiled into single.csv files using custom scripts for further analysis. The 90% confidence intervals and median values of the 500 simulations for the population replacement were calculated as the fraction of GD allele harboring mosquitoes of the total adult population size using Microsoft Excel. Non-overlapping (semi-discrete) generation populations were simulated by adjusting the life history parameters for the MGDrivE simulations (S5 Table). Under the adjusted conditions, the adult population within the mixed population was replaced on a daily basis. As a result, the GD carrying population was replacing the non-GD population every 9 days following initial release in the simulation time given the specified life history parameters. The solutions for the GD carriers and GDBI frequencies were therefore calculated from the values coinciding with the maximal population size within discrete time intervals of 9 days. Generations from the discrete model simulations were matched to the cage trial generation at which average GD invasion reached 50% (G7 for the *nanos*-GD populations and G9 for the *zpg*-GD populations). This was performed to account for some unexpectedly high rates of invasion (i.e., not explicable by GD alone) observed for some of the cage trial populations in the early generations, which may have resulted from stochastic effects due to the low release ratio (Fig 1B). The Metrics package was used in R Studio to calculate the predictive power of the models for the cage trial time-series data. The forecast accuracy of the GD model parameterized with different fitness costs was compared to the observed cage trial allele frequencies by calculating the mean absolute scaled error (MASE) [71].

## Supporting information

S1 Fig Wright-Fisher model output showing loss of an allele introduced at low frequency (2.5%) into populations with different effective sizes. (PPTX)

S2 Fig Recurrent *de novo* gene drive blocking indels. (PPTX)

S3 Fig *nanos*-GD model adjustments considering maternal deposition (M3) and maternal deposition plus fitness cost (M4). (PPTX)

S4 Fig Genotype smoothing function applied to homozygosity. (PPTX)

S1 Table Gene drive carriers by sex. (PPTX)

S2 Table *De novo* gene drive blocking indels (GDBI) observed among cage trial populations. (PPTX)

S3 Table Homing rate parameter measures from single-crosses. (PPTX)

S4 Table Reciprocal gene drive model fit comparison. (PPTX)

S5 Table Life history parameters used for gene drive modeling. (PPTX)

S1 Text Gene drive model, single-locus HEG, with sex-specific homing and maternal deposition, neutral gene drive blocking indels (GDBI). (PPTX)

S2 Text Gene drive specific maternal deposition rates, summary data. (PPTX)

S1 Data Study data underlying the experimental and modeling results. (XLSX)
