# Supplementary material for: Performance of two low-threshold population replacement gene drives in cage populations of the yellow fever mosquito, Aedes aegypti
Source: PLoS Genet. 2025 Jun 26;21(6):e1011757. doi: 10.1371/journal.pgen.1011757 (PMC12221180; doi:10.1371/journal.pgen.1011757)
Supplement: S1 Fig — (PPTX) [file pgen.1011757.s001.pptx]

## Slide 1
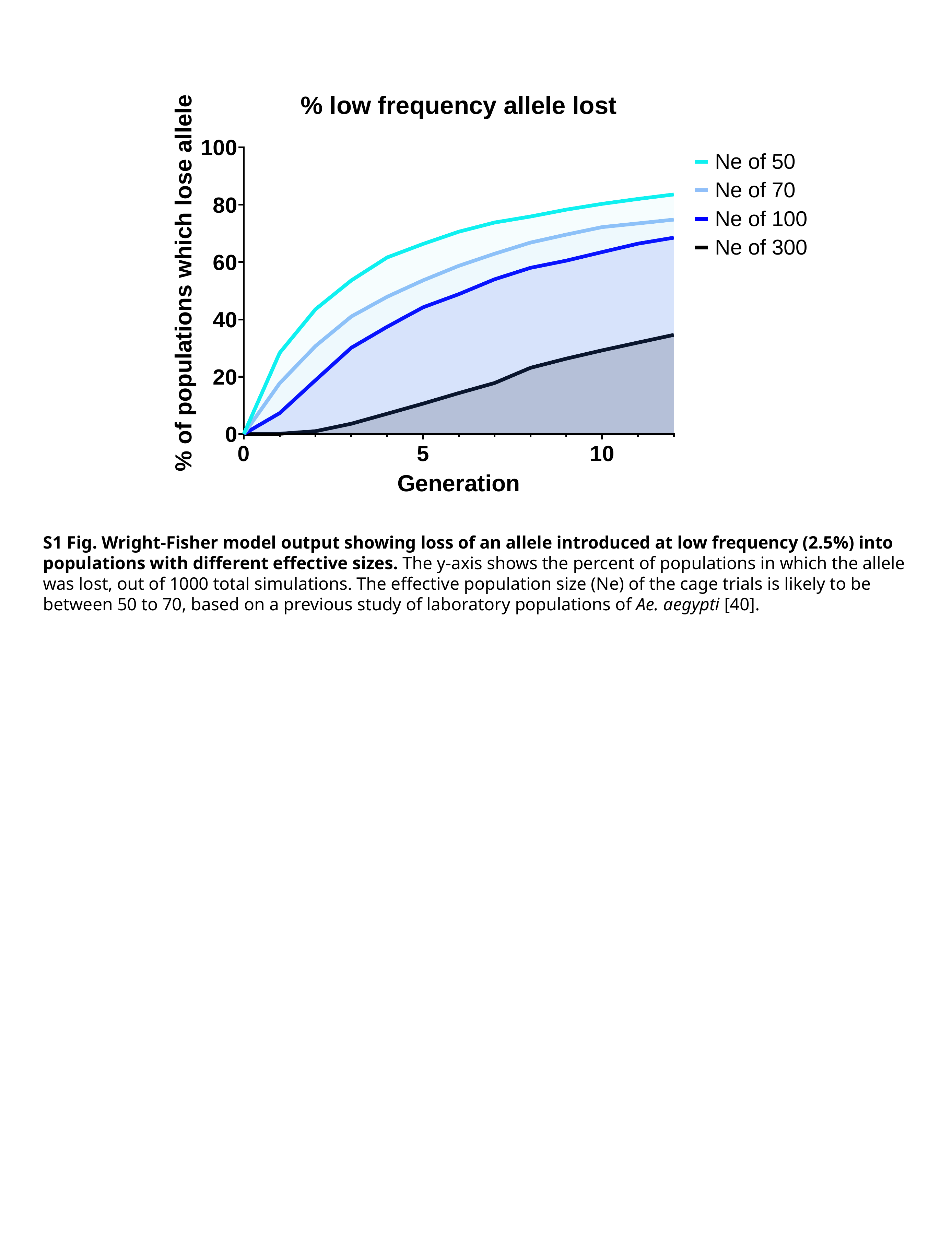

S1 Fig. Wright-Fisher model output showing loss of an allele introduced at low frequency (2.5%) into populations with different effective sizes. The y-axis shows the percent of populations in which the allele was lost, out of 1000 total simulations. The effective population size (Ne) of the cage trials is likely to be between 50 to 70, based on a previous study of laboratory populations of Ae. aegypti [40].
