# Supplementary material for: Performance of two low-threshold population replacement gene drives in cage populations of the yellow fever mosquito, Aedes aegypti
Source: PLoS Genet. 2025 Jun 26;21(6):e1011757. doi: 10.1371/journal.pgen.1011757 (PMC12221180; doi:10.1371/journal.pgen.1011757)
Supplement: S2 Fig — (PPTX) [file pgen.1011757.s002.pptx]

## Slide 1
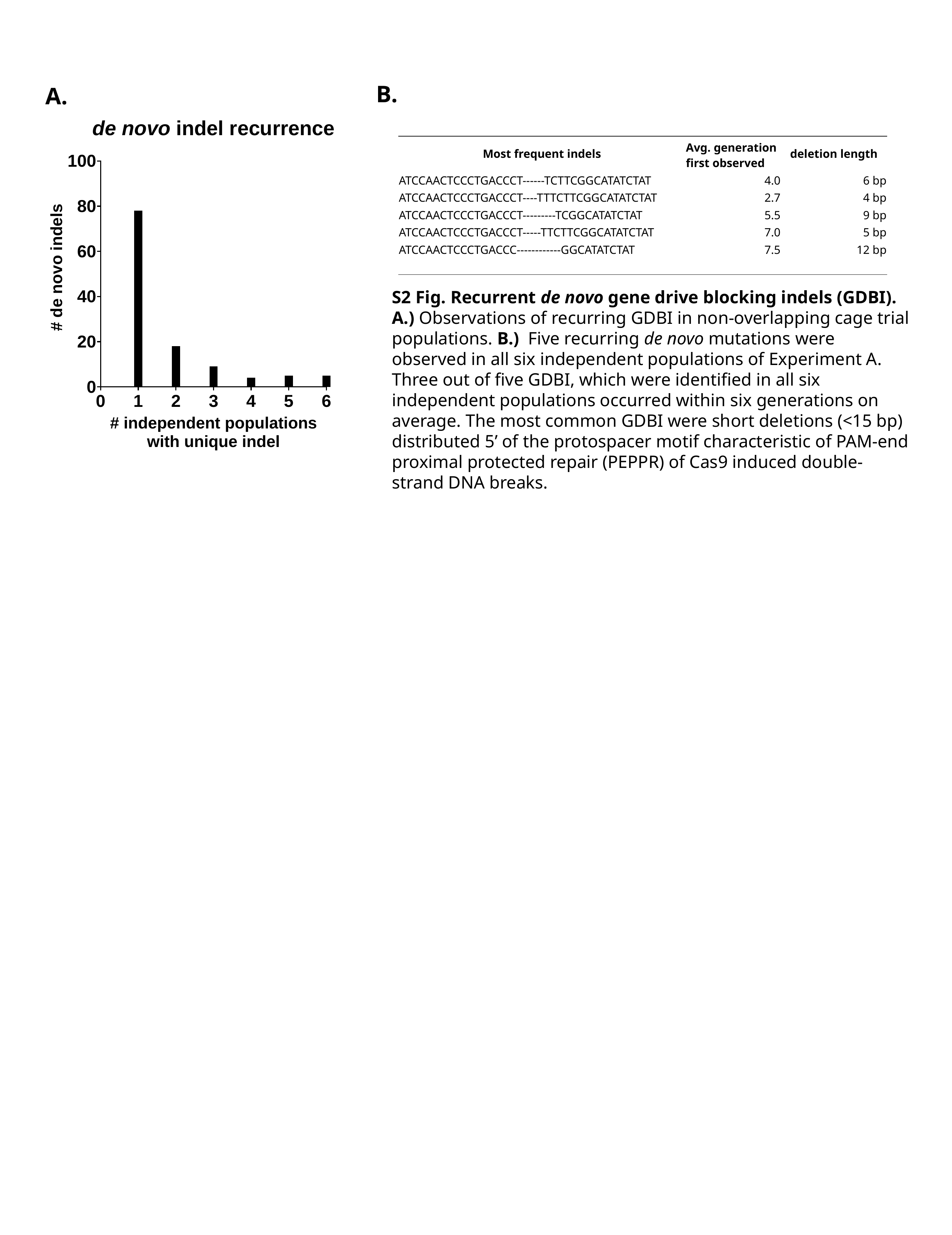

B.
A.
S2 Fig. Recurrent de novo gene drive blocking indels (GDBI). A.) Observations of recurring GDBI in non-overlapping cage trial populations. B.) Five recurring de novo mutations were observed in all six independent populations of Experiment A. Three out of five GDBI, which were identified in all six independent populations occurred within six generations on average. The most common GDBI were short deletions (<15 bp) distributed 5’ of the protospacer motif characteristic of PAM-end proximal protected repair (PEPPR) of Cas9 induced double-strand DNA breaks.
| Most frequent indels | Avg. generation first observed | deletion length |
| --- | --- | --- |
| ATCCAACTCCCTGACCCT------TCTTCGGCATATCTAT | 4.0 | 6 bp |
| ATCCAACTCCCTGACCCT----TTTCTTCGGCATATCTAT | 2.7 | 4 bp |
| ATCCAACTCCCTGACCCT---------TCGGCATATCTAT | 5.5 | 9 bp |
| ATCCAACTCCCTGACCCT-----TTCTTCGGCATATCTAT | 7.0 | 5 bp |
| ATCCAACTCCCTGACCC------------GGCATATCTAT | 7.5 | 12 bp |
| | | |
