# Supplementary material for: Performance of two low-threshold population replacement gene drives in cage populations of the yellow fever mosquito, Aedes aegypti
Source: PLoS Genet. 2025 Jun 26;21(6):e1011757. doi: 10.1371/journal.pgen.1011757 (PMC12221180; doi:10.1371/journal.pgen.1011757)
Supplement: S3 Fig — (PPTX) [file pgen.1011757.s003.pptx]

## Slide 1
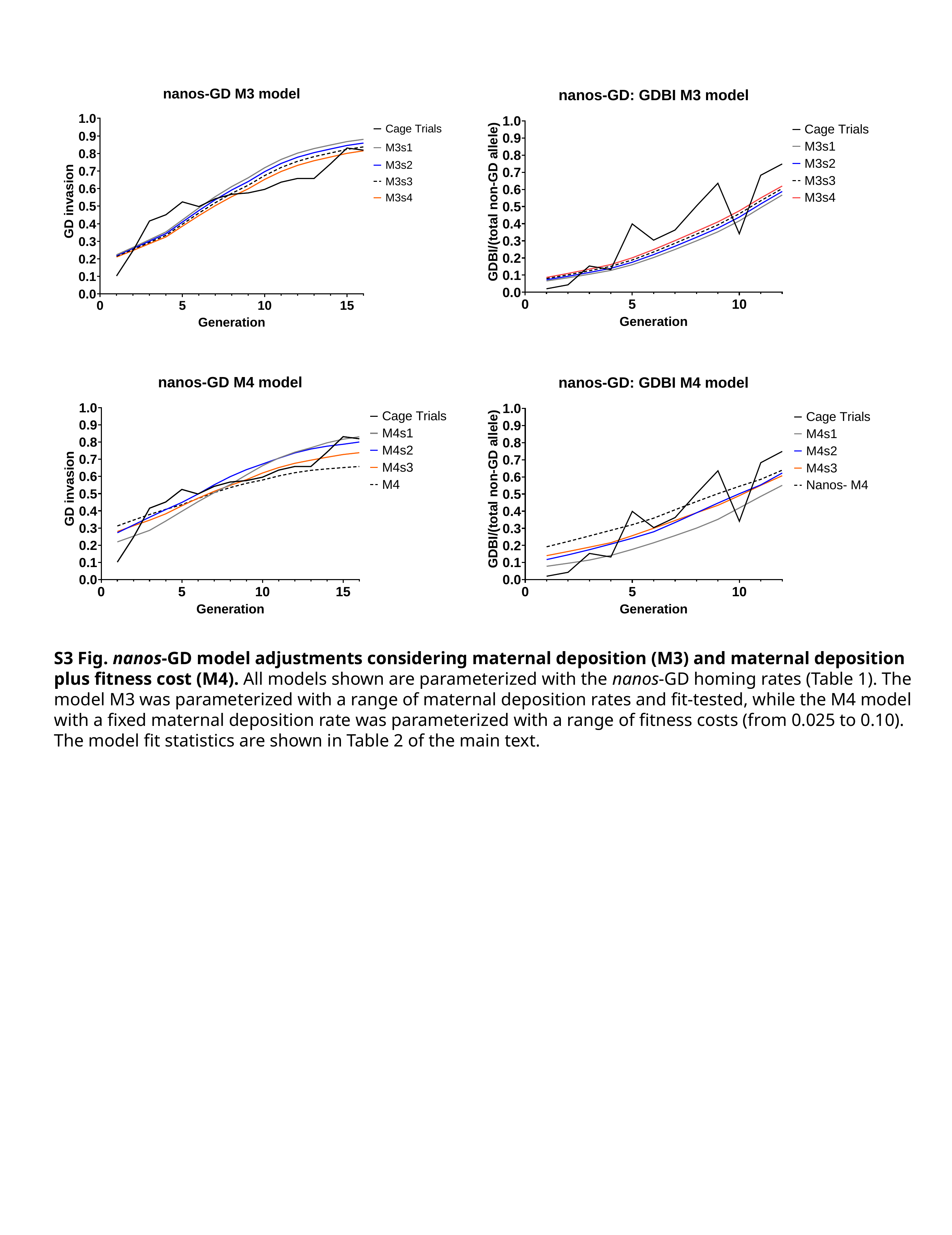

S3 Fig. nanos-GD model adjustments considering maternal deposition (M3) and maternal deposition plus fitness cost (M4). All models shown are parameterized with the nanos-GD homing rates (Table 1). The model M3 was parameterized with a range of maternal deposition rates and fit-tested, while the M4 model with a fixed maternal deposition rate was parameterized with a range of fitness costs (from 0.025 to 0.10). The model fit statistics are shown in Table 2 of the main text.
