# Supplementary material for: Performance of two low-threshold population replacement gene drives in cage populations of the yellow fever mosquito, Aedes aegypti
Source: PLoS Genet. 2025 Jun 26;21(6):e1011757. doi: 10.1371/journal.pgen.1011757 (PMC12221180; doi:10.1371/journal.pgen.1011757)
Supplement: S4 Fig — (PPTX) [file pgen.1011757.s004.pptx]

## Slide 1
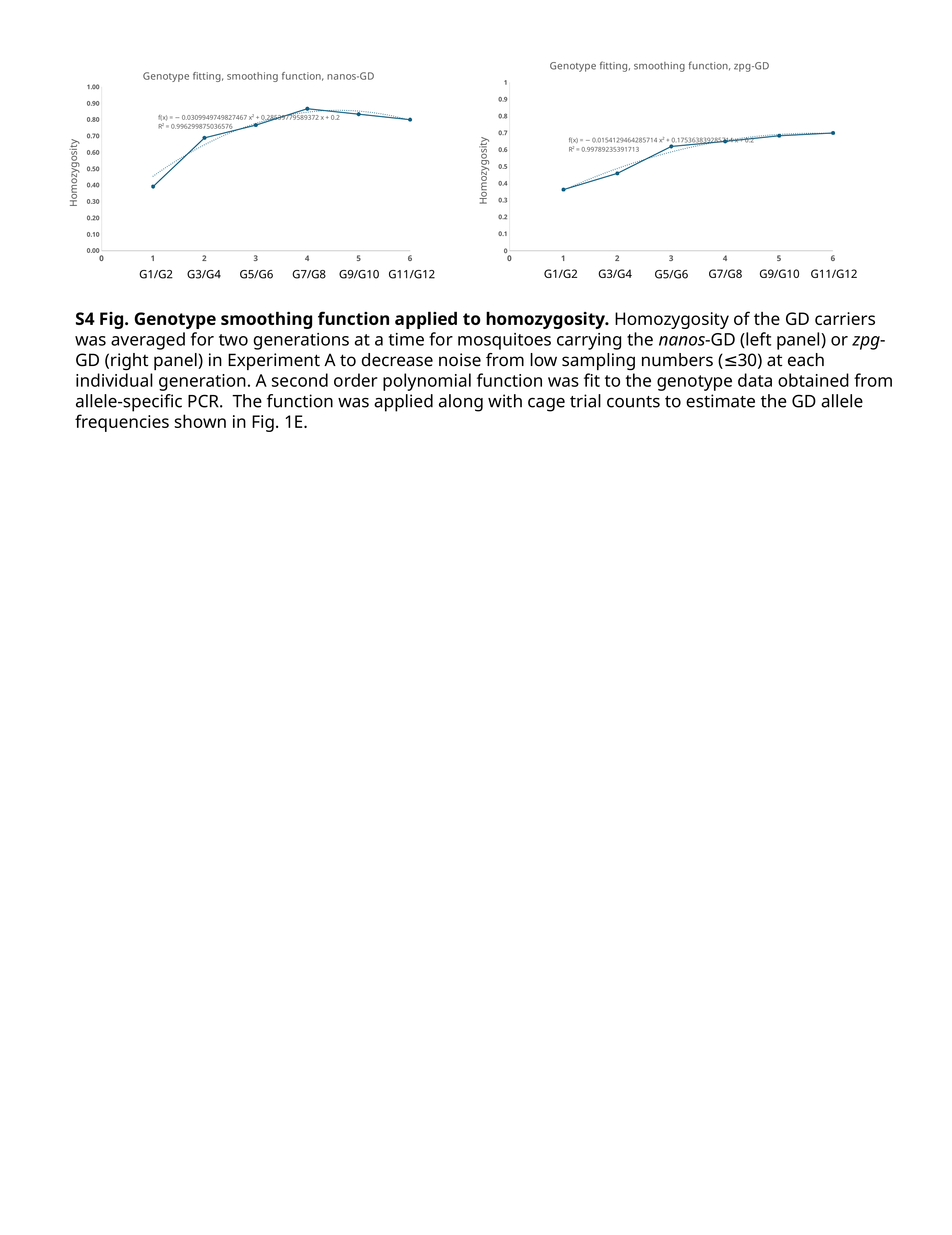

### Chart: Genotype fitting, smoothing function, zpg-GD
| Category | |
|---|---|G7/G8
G9/G10
G11/G12
G1/G2
G3/G4
G5/G6
### Chart: Genotype fitting, smoothing function, nanos-GD
| Category | |
|---|---|G3/G4
G5/G6
G11/G12
G7/G8
G1/G2
G9/G10
S4 Fig. Genotype smoothing function applied to homozygosity. Homozygosity of the GD carriers was averaged for two generations at a time for mosquitoes carrying the nanos-GD (left panel) or zpg-GD (right panel) in Experiment A to decrease noise from low sampling numbers (≤30) at each individual generation. A second order polynomial function was fit to the genotype data obtained from allele-specific PCR. The function was applied along with cage trial counts to estimate the GD allele frequencies shown in Fig. 1E.
