# Supplementary material for: Performance of two low-threshold population replacement gene drives in cage populations of the yellow fever mosquito, Aedes aegypti
Source: PLoS Genet. 2025 Jun 26;21(6):e1011757. doi: 10.1371/journal.pgen.1011757 (PMC12221180; doi:10.1371/journal.pgen.1011757)
Supplement: S1 Table — (PPTX) [file pgen.1011757.s005.pptx]

## Slide 1
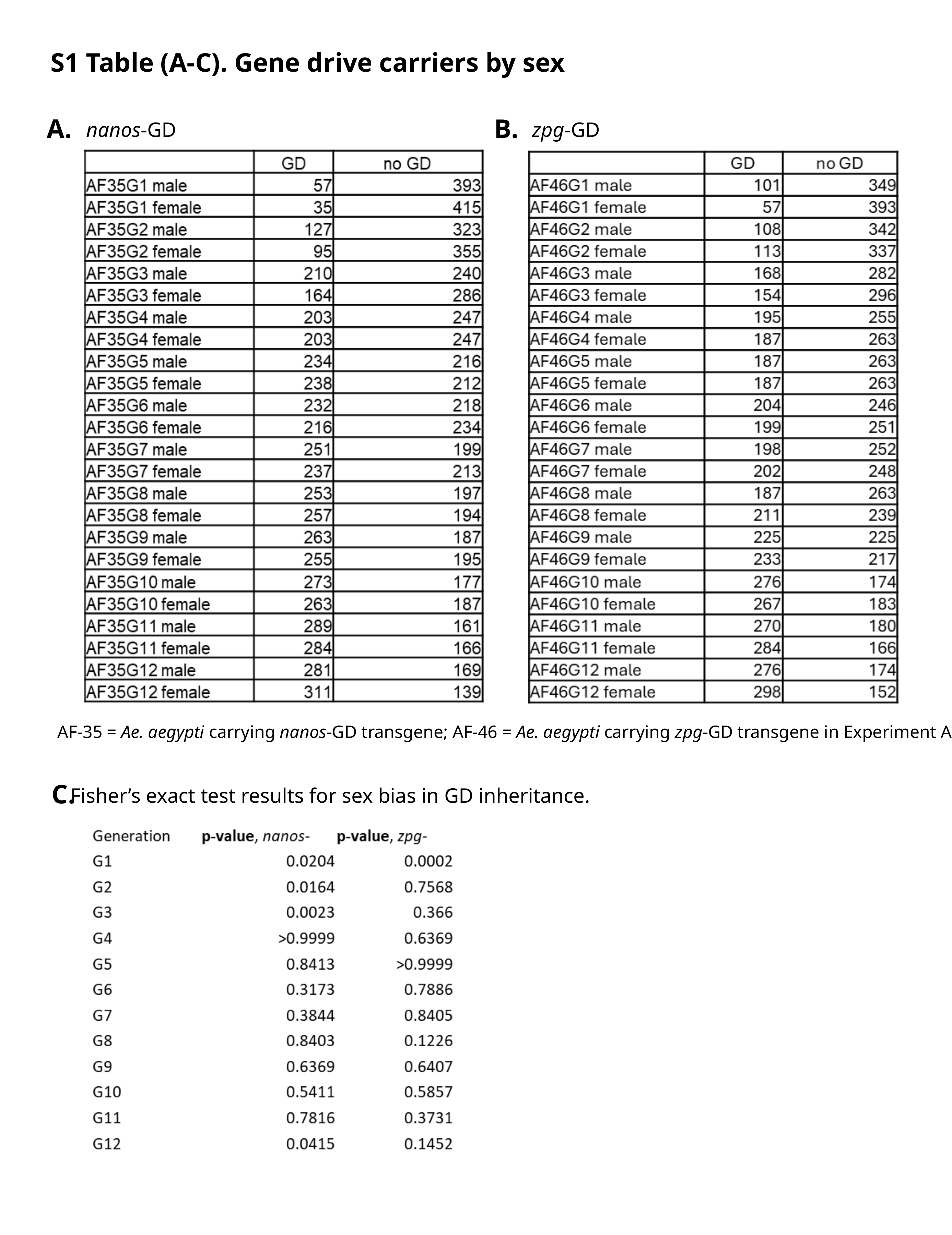

S1 Table (A-C). Gene drive carriers by sex
A.
B.
nanos-GD
zpg-GD
AF-35 = Ae. aegypti carrying nanos-GD transgene; AF-46 = Ae. aegypti carrying zpg-GD transgene in Experiment A.
C.
Fisher’s exact test results for sex bias in GD inheritance.
