# Supplementary material for: Performance of two low-threshold population replacement gene drives in cage populations of the yellow fever mosquito, Aedes aegypti
Source: PLoS Genet. 2025 Jun 26;21(6):e1011757. doi: 10.1371/journal.pgen.1011757 (PMC12221180; doi:10.1371/journal.pgen.1011757)
Supplement: S2 Table — (PPTX) [file pgen.1011757.s006.pptx]

## Slide 1
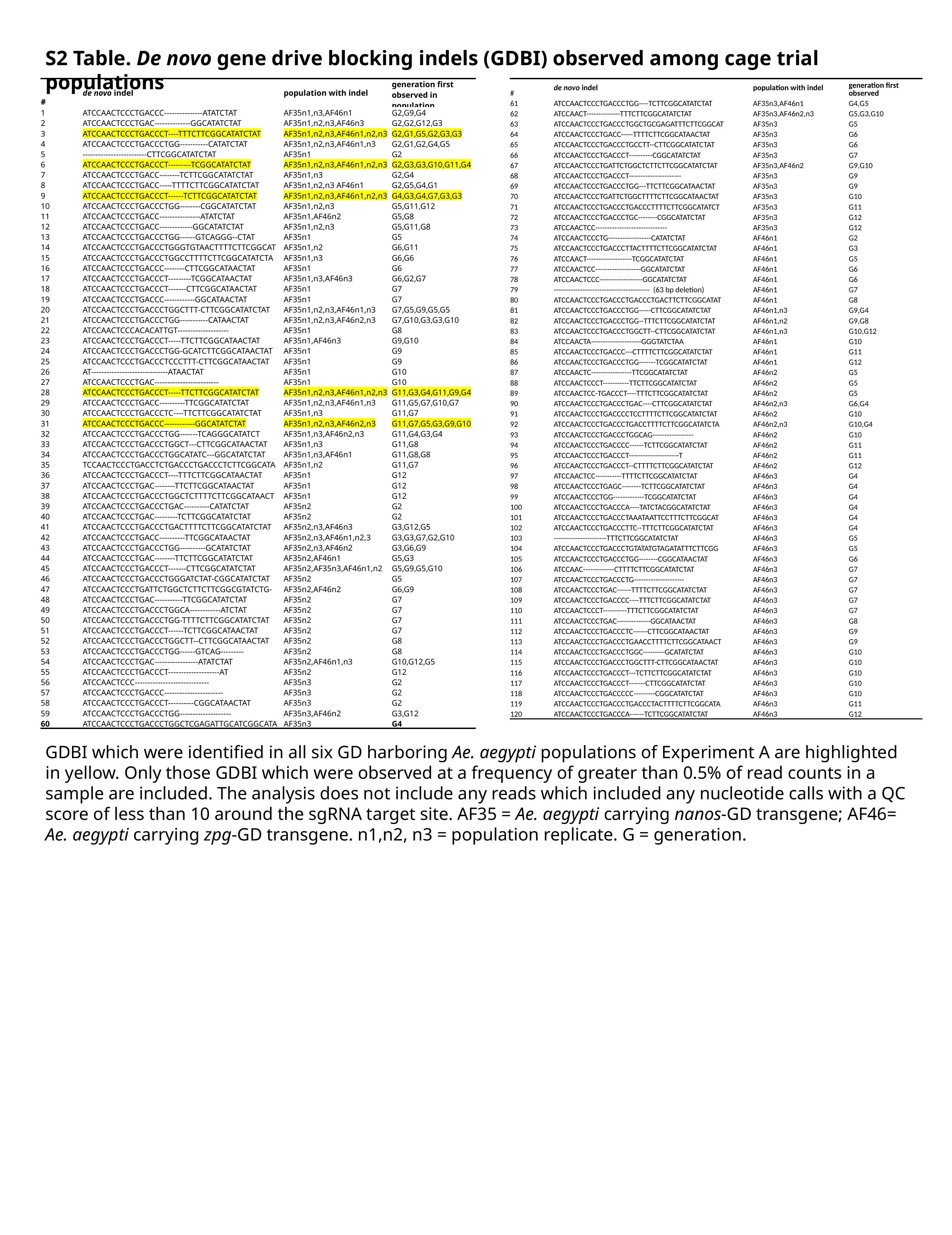

S2 Table. De novo gene drive blocking indels (GDBI) observed among cage trial populations
| # | de novo indel | population with indel | generation first observed in population |
| --- | --- | --- | --- |
| 1 | ATCCAACTCCCTGACCC---------------ATATCTAT | AF35n1,n3,AF46n1 | G2,G9,G4 |
| 2 | ATCCAACTCCCTGAC--------------GGCATATCTAT | AF35n1,n2,n3,AF46n3 | G2,G2,G12,G3 |
| 3 | ATCCAACTCCCTGACCCT----TTTCTTCGGCATATCTAT | AF35n1,n2,n3,AF46n1,n2,n3 | G2,G1,G5,G2,G3,G3 |
| 4 | ATCCAACTCCCTGACCCTGG-----------CATATCTAT | AF35n1,n2,n3,AF46n1,n3 | G2,G1,G2,G4,G5 |
| 5 | -------------------------CTTCGGCATATCTAT | AF35n1 | G2 |
| 6 | ATCCAACTCCCTGACCCT---------TCGGCATATCTAT | AF35n1,n2,n3,AF46n1,n2,n3 | G2,G3,G3,G10,G11,G4 |
| 7 | ATCCAACTCCCTGACC--------TCTTCGGCATATCTAT | AF35n1,n3 | G2,G4 |
| 8 | ATCCAACTCCCTGACC-----TTTTCTTCGGCATATCTAT | AF35n1,n2,n3 AF46n1 | G2,G5,G4,G1 |
| 9 | ATCCAACTCCCTGACCCT------TCTTCGGCATATCTAT | AF35n1,n2,n3,AF46n1,n2,n3 | G4,G3,G4,G7,G3,G3 |
| 10 | ATCCAACTCCCTGACCCTGG--------CGGCATATCTAT | AF35n1,n2,n3 | G5,G11,G12 |
| 11 | ATCCAACTCCCTGACC----------------ATATCTAT | AF35n1,AF46n2 | G5,G8 |
| 12 | ATCCAACTCCCTGACC-------------GGCATATCTAT | AF35n1,n2,n3 | G5,G11,G8 |
| 13 | ATCCAACTCCCTGACCCTGG------GTCAGGG--CTAT | AF35n1 | G5 |
| 14 | ATCCAACTCCCTGACCCTGGGTGTAACTTTTCTTCGGCAT | AF35n1,n2 | G6,G11 |
| 15 | ATCCAACTCCCTGACCCTGGCCTTTTCTTCGGCATATCTA | AF35n1,n3 | G6,G6 |
| 16 | ATCCAACTCCCTGACCC--------CTTCGGCATAACTAT | AF35n1 | G6 |
| 17 | ATCCAACTCCCTGACCCT---------TCGGCATAACTAT | AF35n1,n3,AF46n3 | G6,G2,G7 |
| 18 | ATCCAACTCCCTGACCCT-------CTTCGGCATAACTAT | AF35n1 | G7 |
| 19 | ATCCAACTCCCTGACCC------------GGCATAACTAT | AF35n1 | G7 |
| 20 | ATCCAACTCCCTGACCCTGGCTTT-CTTCGGCATATCTAT | AF35n1,n2,n3,AF46n1,n3 | G7,G5,G9,G5,G5 |
| 21 | ATCCAACTCCCTGACCCTGG-----------CATAACTAT | AF35n1,n2,n3,AF46n2,n3 | G7,G10,G3,G3,G10 |
| 22 | ATCCAACTCCCACACATTGT-------------------- | AF35n1 | G8 |
| 23 | ATCCAACTCCCTGACCCT-----TTCTTCGGCATAACTAT | AF35n1,AF46n3 | G9,G10 |
| 24 | ATCCAACTCCCTGACCCTGG-GCATCTTCGGCATAACTAT | AF35n1 | G9 |
| 25 | ATCCAACTCCCTGACCCTCCCTTT-CTTCGGCATAACTAT | AF35n1 | G9 |
| 26 | AT------------------------------ATAACTAT | AF35n1 | G10 |
| 27 | ATCCAACTCCCTGAC------------------------- | AF35n1 | G10 |
| 28 | ATCCAACTCCCTGACCCT-----TTCTTCGGCATATCTAT | AF35n1,n2,n3,AF46n1,n2,n3 | G11,G3,G4,G11,G9,G4 |
| 29 | ATCCAACTCCCTGACC----------TTCGGCATATCTAT | AF35n1,n2,n3,AF46n1,n3 | G11,G5,G7,G10,G7 |
| 30 | ATCCAACTCCCTGACCCTC----TTCTTCGGCATATCTAT | AF35n1,n3 | G11,G7 |
| 31 | ATCCAACTCCCTGACCC------------GGCATATCTAT | AF35n1,n2,n3,AF46n2,n3 | G11,G7,G5,G3,G9,G10 |
| 32 | ATCCAACTCCCTGACCCTGG-------TCAGGGCATATCT | AF35n1,n3,AF46n2,n3 | G11,G4,G3,G4 |
| 33 | ATCCAACTCCCTGACCCTGGCT---CTTCGGCATAACTAT | AF35n1,n3 | G11,G8 |
| 34 | ATCCAACTCCCTGACCCTGGCATATC---GGCATATCTAT | AF35n1,n3,AF46n1 | G11,G8,G8 |
| 35 | TCCAACTCCCTGACCTCTGACCCTGACCCTCTTCGGCATA | AF35n1,n2 | G11,G7 |
| 36 | ATCCAACTCCCTGACCCT----TTTCTTCGGCATAACTAT | AF35n1 | G12 |
| 37 | ATCCAACTCCCTGAC--------TTCTTCGGCATAACTAT | AF35n1 | G12 |
| 38 | ATCCAACTCCCTGACCCTGGCTCTTTTCTTCGGCATAACT | AF35n1 | G12 |
| 39 | ATCCAACTCCCTGACCCTGAC----------CATATCTAT | AF35n2 | G2 |
| 40 | ATCCAACTCCCTGAC---------TCTTCGGCATATCTAT | AF35n2 | G2 |
| 41 | ATCCAACTCCCTGACCCTGACTTTTCTTCGGCATATCTAT | AF35n2,n3,AF46n3 | G3,G12,G5 |
| 42 | ATCCAACTCCCTGACC----------TTCGGCATAACTAT | AF35n2,n3,AF46n1,n2,3 | G3,G3,G7,G2,G10 |
| 43 | ATCCAACTCCCTGACCCTGG----------GCATATCTAT | AF35n2,n3,AF46n2 | G3,G6,G9 |
| 44 | ATCCAACTCCCTGAC--------TTCTTCGGCATATCTAT | AF35n2,AF46n1 | G5,G3 |
| 45 | ATCCAACTCCCTGACCCT-------CTTCGGCATATCTAT | AF35n2,AF35n3,AF46n1,n2 | G5,G9,G5,G10 |
| 46 | ATCCAACTCCCTGACCCTGGGATCTAT-CGGCATATCTAT | AF35n2 | G5 |
| 47 | ATCCAACTCCCTGATTCTGGCTCTTCTTCGGCGTATCTG- | AF35n2,AF46n2 | G6,G9 |
| 48 | ATCCAACTCCCTGAC-----------TTCGGCATATCTAT | AF35n2 | G7 |
| 49 | ATCCAACTCCCTGACCCTGGCA------------ATCTAT | AF35n2 | G7 |
| 50 | ATCCAACTCCCTGACCCTGG-TTTTCTTCGGCATATCTAT | AF35n2 | G7 |
| 51 | ATCCAACTCCCTGACCCT------TCTTCGGCATAACTAT | AF35n2 | G7 |
| 52 | ATCCAACTCCCTGACCCTGGCTT--CTTCGGCATAACTAT | AF35n2 | G8 |
| 53 | ATCCAACTCCCTGACCCTGG------GTCAG--------- | AF35n2 | G8 |
| 54 | ATCCAACTCCCTGAC-----------------ATATCTAT | AF35n2,AF46n1,n3 | G10,G12,G5 |
| 55 | ATCCAACTCCCTGACCCT--------------------AT | AF35n2 | G12 |
| 56 | ATCCAACTCCC----------------------------- | AF35n3 | G2 |
| 57 | ATCCAACTCCCTGACCC----------------------- | AF35n3 | G2 |
| 58 | ATCCAACTCCCTGACCCT----------CGGCATAACTAT | AF35n3 | G2 |
| 59 | ATCCAACTCCCTGACCCTGG-------------------- | AF35n3,AF46n2 | G3,G12 |
| 60 | ATCCAACTCCCTGACCCTGGCTCGAGATTGCATCGGCATA | AF35n3 | G4 |
| # | de novo indel | population with indel | generation first observed |
| --- | --- | --- | --- |
| 61 | ATCCAACTCCCTGACCCTGG----TCTTCGGCATATCTAT | AF35n3,AF46n1 | G4,G5 |
| 62 | ATCCAACT--------------TTTCTTCGGCATATCTAT | AF35n3,AF46n2,n3 | G5,G3,G10 |
| 63 | ATCCAACTCCCTGACCCTGGCTGCGAGATTTCTTCGGCAT | AF35n3 | G5 |
| 64 | ATCCAACTCCCTGACC-----TTTTCTTCGGCATAACTAT | AF35n3 | G6 |
| 65 | ATCCAACTCCCTGACCCTGCCTT--CTTCGGCATATCTAT | AF35n3 | G6 |
| 66 | ATCCAACTCCCTGACCCT----------CGGCATATCTAT | AF35n3 | G7 |
| 67 | ATCCAACTCCCTGATTCTGGCTCTTCTTCGGCATATCTAT | AF35n3,AF46n2 | G9,G10 |
| 68 | ATCCAACTCCCTGACCCT---------------------- | AF35n3 | G9 |
| 69 | ATCCAACTCCCTGACCCTGG---TTCTTCGGCATAACTAT | AF35n3 | G9 |
| 70 | ATCCAACTCCCTGATTCTGGCTTTTCTTCGGCATAACTAT | AF35n3 | G10 |
| 71 | ATCCAACTCCCTGACCCTGACCCTTTTCTTCGGCATATCT | AF35n3 | G11 |
| 72 | ATCCAACTCCCTGACCCTGC--------CGGCATATCTAT | AF35n3 | G12 |
| 73 | ATCCAACTCC------------------------------ | AF35n3 | G12 |
| 74 | ATCCAACTCCCTG------------------CATATCTAT | AF46n1 | G2 |
| 75 | ATCCAACTCCCTGACCCTTACTTTTCTTCGGCATATCTAT | AF46n1 | G3 |
| 76 | ATCCAACT-------------------TCGGCATATCTAT | AF46n1 | G5 |
| 77 | ATCCAACTCC-------------------GGCATATCTAT | AF46n1 | G6 |
| 78 | ATCCAACTCCC------------------GGCATATCTAT | AF46n1 | G6 |
| 79 | ---------------------------------------- (63 bp deletion) | AF46n1 | G7 |
| 80 | ATCCAACTCCCTGACCCTGACCCTGACTTCTTCGGCATAT | AF46n1 | G8 |
| 81 | ATCCAACTCCCTGACCCTGG-----CTTCGGCATATCTAT | AF46n1,n3 | G9,G4 |
| 82 | ATCCAACTCCCTGACCCTGG--TTTCTTCGGCATATCTAT | AF46n1,n2 | G9,G8 |
| 83 | ATCCAACTCCCTGACCCTGGCTT--CTTCGGCATATCTAT | AF46n1,n3 | G10,G12 |
| 84 | ATCCAACTA---------------------GGGTATCTAA | AF46n1 | G10 |
| 85 | ATCCAACTCCCTGACCC---CTTTTCTTCGGCATATCTAT | AF46n1 | G11 |
| 86 | ATCCAACTCCCTGACCCTGG-------TCGGCATATCTAT | AF46n1 | G12 |
| 87 | ATCCAACTC-----------------TTCGGCATATCTAT | AF46n2 | G5 |
| 88 | ATCCAACTCCCT-----------TTCTTCGGCATATCTAT | AF46n2 | G5 |
| 89 | ATCCAACTCC-TGACCCT----TTTCTTCGGCATATCTAT | AF46n2 | G5 |
| 90 | ATCCAACTCCCTGACCCTGAC----CTTCGGCATATCTAT | AF46n2,n3 | G6,G4 |
| 91 | ATCCAACTCCCTGACCCCTCCTTTTCTTCGGCATATCTAT | AF46n2 | G10 |
| 92 | ATCCAACTCCCTGACCCTGACCTTTTCTTCGGCATATCTA | AF46n2,n3 | G10,G4 |
| 93 | ATCCAACTCCCTGACCCTGGCAG----------------- | AF46n2 | G10 |
| 94 | ATCCAACTCCCTGACCCC------TCTTCGGCATATCTAT | AF46n2 | G11 |
| 95 | ATCCAACTCCCTGACCCT---------------------T | AF46n2 | G11 |
| 96 | ATCCAACTCCCTGACCCT--CTTTTCTTCGGCATATCTAT | AF46n2 | G12 |
| 97 | ATCCAACTCC-----------TTTTCTTCGGCATATCTAT | AF46n3 | G4 |
| 98 | ATCCAACTCCCTGAGC--------TCTTCGGCATATCTAT | AF46n3 | G4 |
| 99 | ATCCAACTCCCTGG-------------TCGGCATATCTAT | AF46n3 | G4 |
| 100 | ATCCAACTCCCTGACCCA----TATCTACGGCATATCTAT | AF46n3 | G4 |
| 101 | ATCCAACTCCCTGACCCTAAATAATTCCTTTCTTCGGCAT | AF46n3 | G4 |
| 102 | ATCCAACTCCCTGACCCTTC--TTTCTTCGGCATATCTAT | AF46n3 | G4 |
| 103 | ----------------------TTTCTTCGGCATATCTAT | AF46n3 | G5 |
| 104 | ATCCAACTCCCTGACCCTGTATATGTAGATATTTCTTCGG | AF46n3 | G5 |
| 105 | ATCCAACTCCCTGACCCTGG--------CGGCATAACTAT | AF46n3 | G6 |
| 106 | ATCCAAC-------------CTTTTCTTCGGCATATCTAT | AF46n3 | G7 |
| 107 | ATCCAACTCCCTGACCCTG--------------------- | AF46n3 | G7 |
| 108 | ATCCAACTCCCTGAC------TTTTCTTCGGCATATCTAT | AF46n3 | G7 |
| 109 | ATCCAACTCCCTGACCCC----TTTCTTCGGCATATCTAT | AF46n3 | G7 |
| 110 | ATCCAACTCCCT----------TTTCTTCGGCATATCTAT | AF46n3 | G7 |
| 111 | ATCCAACTCCCTGAC--------------GGCATAACTAT | AF46n3 | G8 |
| 112 | ATCCAACTCCCTGACCCTC------CTTCGGCATAACTAT | AF46n3 | G9 |
| 113 | ATCCAACTCCCTGACCCTGAACCTTTTCTTCGGCATAACT | AF46n3 | G9 |
| 114 | ATCCAACTCCCTGACCCTGGC---------GCATATCTAT | AF46n3 | G10 |
| 115 | ATCCAACTCCCTGACCCTGGCTTT-CTTCGGCATAACTAT | AF46n3 | G10 |
| 116 | ATCCAACTCCCTGACCCT---TCTTCTTCGGCATATCTAT | AF46n3 | G10 |
| 117 | ATCCAACTCCCTGACCCT-------CTTCGGCATATCTAT | AF46n3 | G10 |
| 118 | ATCCAACTCCCTGACCCCC---------CGGCATATCTAT | AF46n3 | G10 |
| 119 | ATCCAACTCCCTGACCCTGACCCTACTTTTCTTCGGCATA | AF46n3 | G11 |
| 120 | ATCCAACTCCCTGACCCA------TCTTCGGCATATCTAT | AF46n3 | G12 |
GDBI which were identified in all six GD harboring Ae. aegypti populations of Experiment A are highlighted in yellow. Only those GDBI which were observed at a frequency of greater than 0.5% of read counts in a sample are included. The analysis does not include any reads which included any nucleotide calls with a QC score of less than 10 around the sgRNA target site. AF35 = Ae. aegypti carrying nanos-GD transgene; AF46= Ae. aegypti carrying zpg-GD transgene. n1,n2, n3 = population replicate. G = generation.
