# Supplementary material for: Performance of two low-threshold population replacement gene drives in cage populations of the yellow fever mosquito, Aedes aegypti
Source: PLoS Genet. 2025 Jun 26;21(6):e1011757. doi: 10.1371/journal.pgen.1011757 (PMC12221180; doi:10.1371/journal.pgen.1011757)
Supplement: S3 Table — (PPTX) [file pgen.1011757.s007.pptx]

## Slide 1
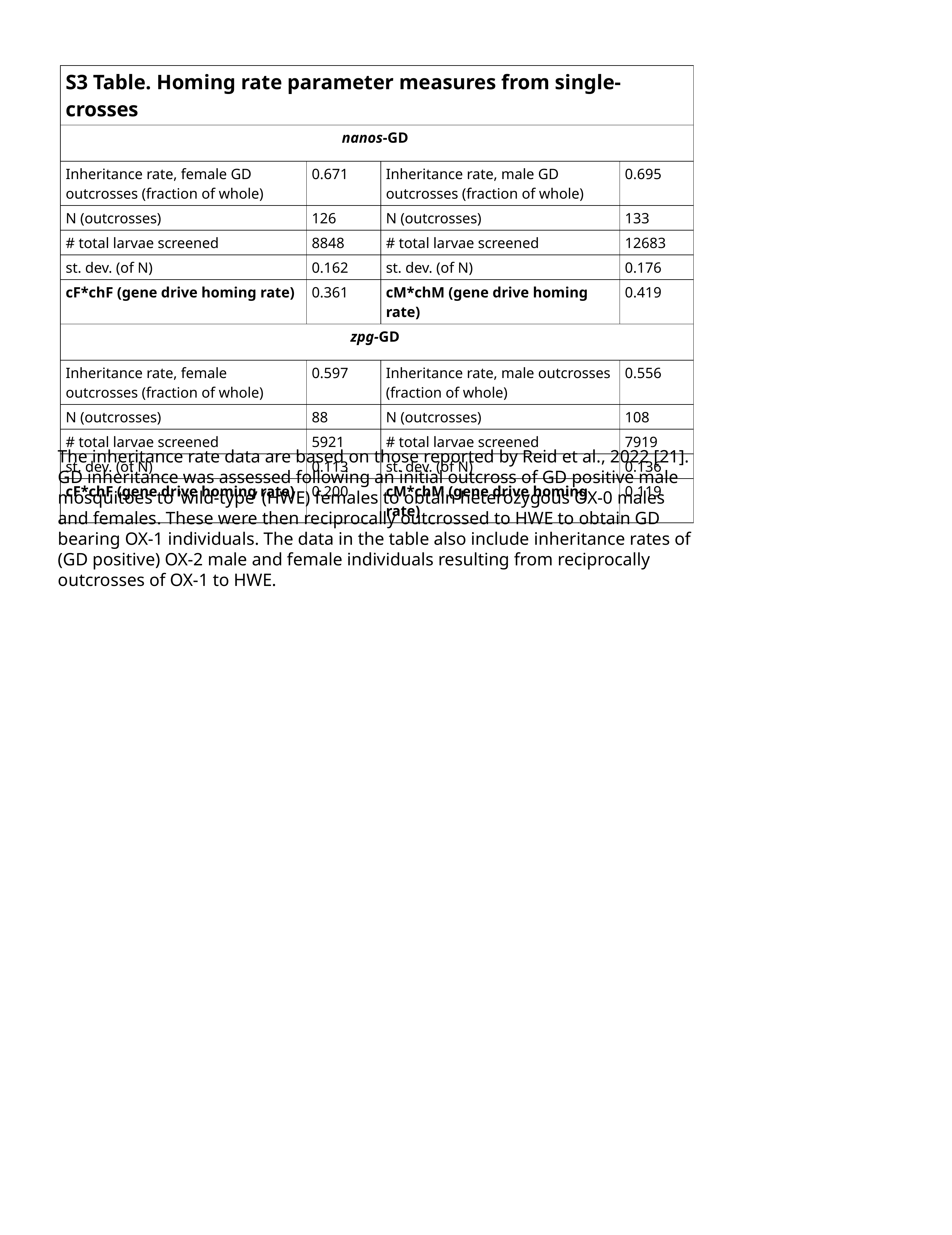

| S3 Table. Homing rate parameter measures from single-crosses | | | |
| --- | --- | --- | --- |
| nanos-GD | | | |
| Inheritance rate, female GD outcrosses (fraction of whole) | 0.671 | Inheritance rate, male GD outcrosses (fraction of whole) | 0.695 |
| N (outcrosses) | 126 | N (outcrosses) | 133 |
| # total larvae screened | 8848 | # total larvae screened | 12683 |
| st. dev. (of N) | 0.162 | st. dev. (of N) | 0.176 |
| cF\*chF (gene drive homing rate) | 0.361 | cM\*chM (gene drive homing rate) | 0.419 |
| zpg-GD | | | |
| Inheritance rate, female outcrosses (fraction of whole) | 0.597 | Inheritance rate, male outcrosses (fraction of whole) | 0.556 |
| N (outcrosses) | 88 | N (outcrosses) | 108 |
| # total larvae screened | 5921 | # total larvae screened | 7919 |
| st. dev. (of N) | 0.113 | st. dev. (of N) | 0.136 |
| cF\*chF (gene drive homing rate) | 0.200 | cM\*chM (gene drive homing rate) | 0.119 |
The inheritance rate data are based on those reported by Reid et al., 2022 [21]. GD inheritance was assessed following an initial outcross of GD positive male mosquitoes to ‘wild-type’ (HWE) females to obtain heterozygous OX-0 males and females. These were then reciprocally outcrossed to HWE to obtain GD bearing OX-1 individuals. The data in the table also include inheritance rates of (GD positive) OX-2 male and female individuals resulting from reciprocally outcrosses of OX-1 to HWE.
