# Supplementary material for: Performance of two low-threshold population replacement gene drives in cage populations of the yellow fever mosquito, Aedes aegypti
Source: PLoS Genet. 2025 Jun 26;21(6):e1011757. doi: 10.1371/journal.pgen.1011757 (PMC12221180; doi:10.1371/journal.pgen.1011757)
Supplement: S4 Table — (PPTX) [file pgen.1011757.s008.pptx]

## Slide 1
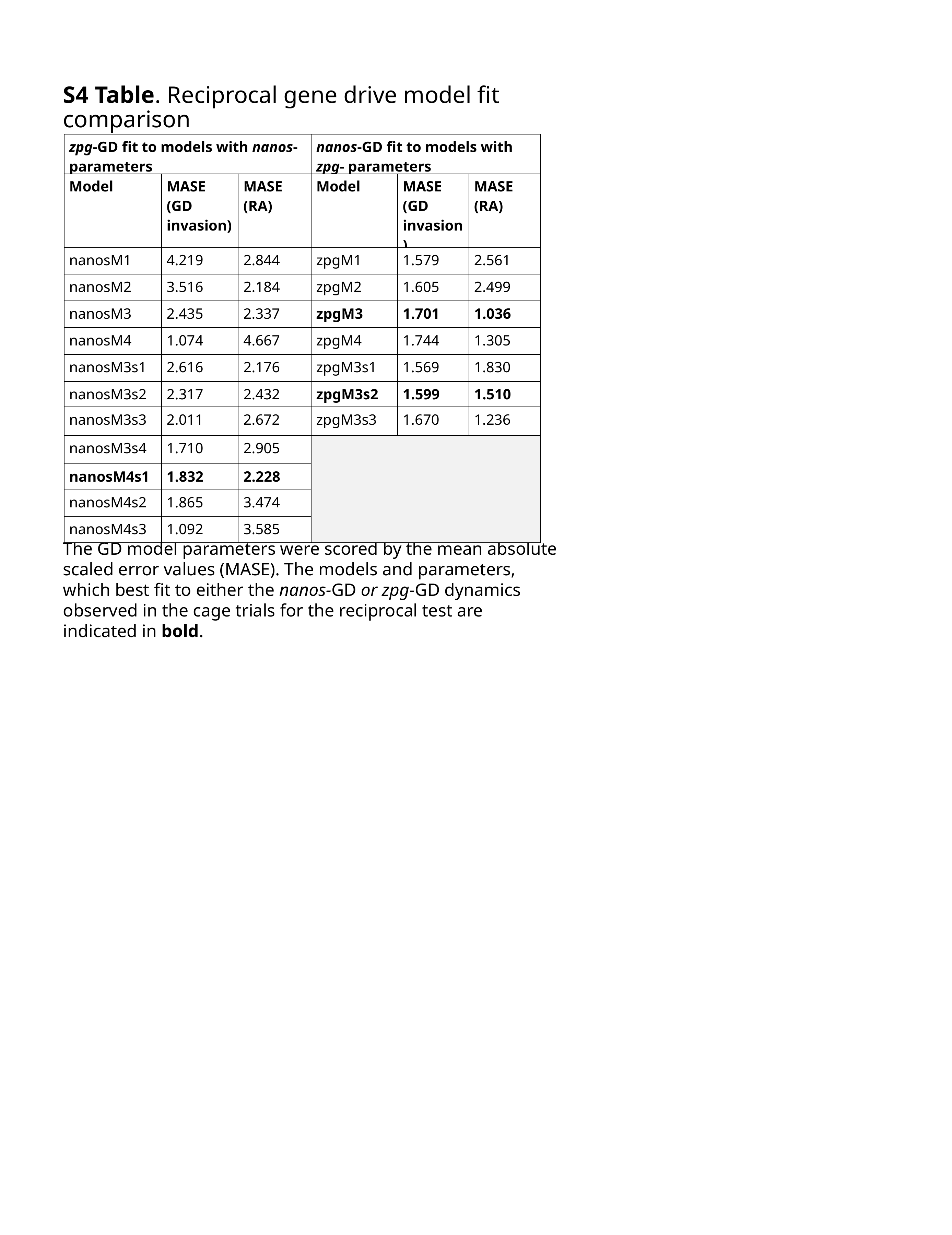

S4 Table. Reciprocal gene drive model fit comparison
| zpg-GD fit to models with nanos- parameters | | | nanos-GD fit to models with zpg- parameters | | |
| --- | --- | --- | --- | --- | --- |
| Model | MASE (GD invasion) | MASE (RA) | Model | MASE (GD invasion) | MASE (RA) |
| nanosM1 | 4.219 | 2.844 | zpgM1 | 1.579 | 2.561 |
| nanosM2 | 3.516 | 2.184 | zpgM2 | 1.605 | 2.499 |
| nanosM3 | 2.435 | 2.337 | zpgM3 | 1.701 | 1.036 |
| nanosM4 | 1.074 | 4.667 | zpgM4 | 1.744 | 1.305 |
| nanosM3s1 | 2.616 | 2.176 | zpgM3s1 | 1.569 | 1.830 |
| nanosM3s2 | 2.317 | 2.432 | zpgM3s2 | 1.599 | 1.510 |
| nanosM3s3 | 2.011 | 2.672 | zpgM3s3 | 1.670 | 1.236 |
| nanosM3s4 | 1.710 | 2.905 | | | |
| nanosM4s1 | 1.832 | 2.228 | | | |
| nanosM4s2 | 1.865 | 3.474 | | | |
| nanosM4s3 | 1.092 | 3.585 | | | |
The GD model parameters were scored by the mean absolute scaled error values (MASE). The models and parameters, which best fit to either the nanos-GD or zpg-GD dynamics observed in the cage trials for the reciprocal test are indicated in bold.
