# Supplementary material for: Performance of two low-threshold population replacement gene drives in cage populations of the yellow fever mosquito, Aedes aegypti
Source: PLoS Genet. 2025 Jun 26;21(6):e1011757. doi: 10.1371/journal.pgen.1011757 (PMC12221180; doi:10.1371/journal.pgen.1011757)
Supplement: S5 Table — (PPTX) [file pgen.1011757.s009.pptx]

## Slide 1
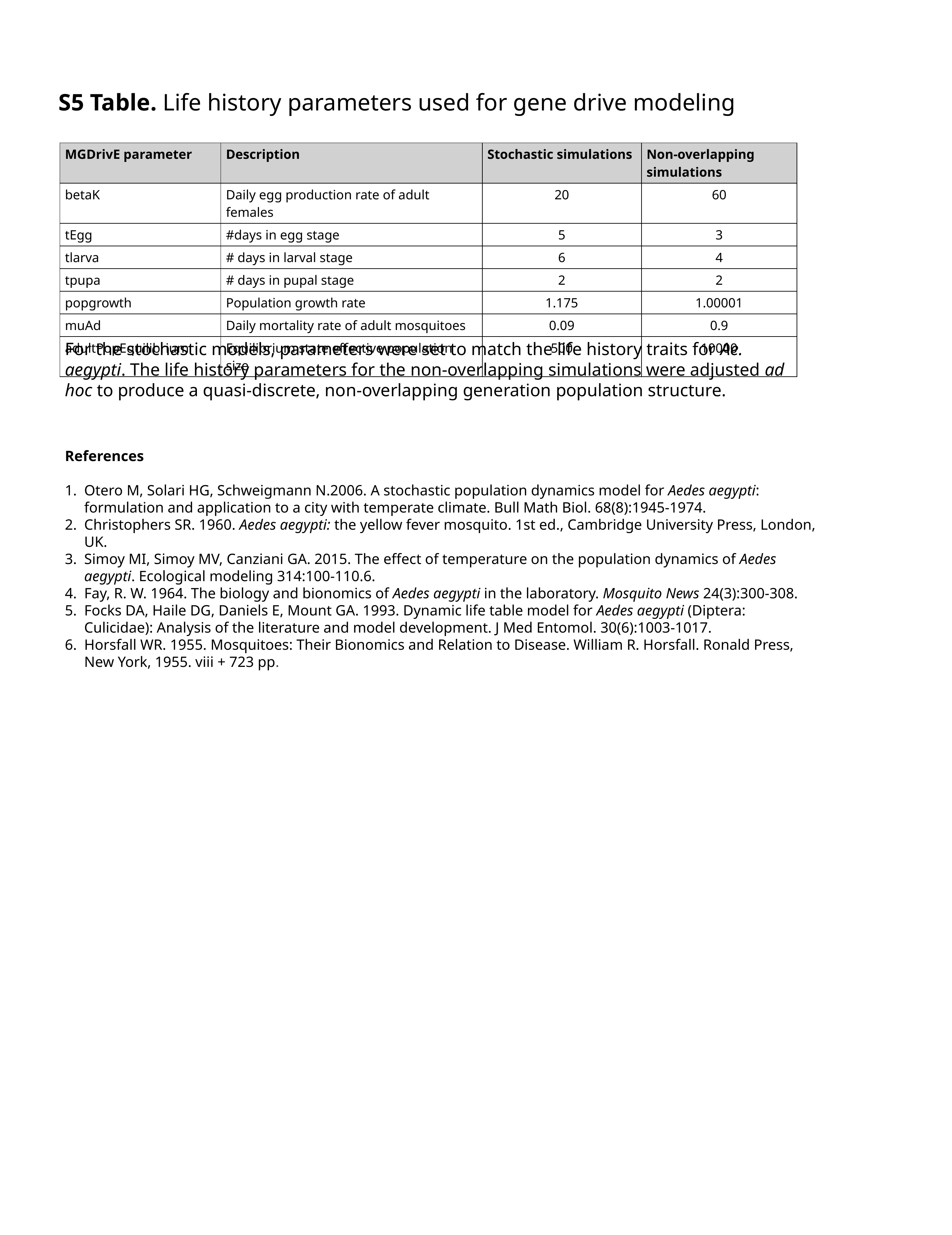

S5 Table. Life history parameters used for gene drive modeling
| MGDrivE parameter | Description | Stochastic simulations | Non-overlapping simulations |
| --- | --- | --- | --- |
| betaK | Daily egg production rate of adult females | 20 | 60 |
| tEgg | #days in egg stage | 5 | 3 |
| tlarva | # days in larval stage | 6 | 4 |
| tpupa | # days in pupal stage | 2 | 2 |
| popgrowth | Population growth rate | 1.175 | 1.00001 |
| muAd | Daily mortality rate of adult mosquitoes | 0.09 | 0.9 |
| adultPopEquilibrium | Equilibrium state effective population size | 500 | 10000 |
For the stochastic models, parameters were set to match the life history traits for Ae. aegypti. The life history parameters for the non-overlapping simulations were adjusted ad hoc to produce a quasi-discrete, non-overlapping generation population structure.
References
Otero M, Solari HG, Schweigmann N.2006. A stochastic population dynamics model for Aedes aegypti: formulation and application to a city with temperate climate. Bull Math Biol. 68(8):1945-1974.
Christophers SR. 1960. Aedes aegypti: the yellow fever mosquito. 1st ed., Cambridge University Press, London, UK.
Simoy MI, Simoy MV, Canziani GA. 2015. The effect of temperature on the population dynamics of Aedes aegypti. Ecological modeling 314:100-110.6.
Fay, R. W. 1964. The biology and bionomics of Aedes aegypti in the laboratory. Mosquito News 24(3):300-308.
Focks DA, Haile DG, Daniels E, Mount GA. 1993. Dynamic life table model for Aedes aegypti (Diptera: Culicidae): Analysis of the literature and model development. J Med Entomol. 30(6):1003-1017.
Horsfall WR. 1955. Mosquitoes: Their Bionomics and Relation to Disease. William R. Horsfall. Ronald Press, New York, 1955. viii + 723 pp.
