# Supplementary material for: Performance of two low-threshold population replacement gene drives in cage populations of the yellow fever mosquito, Aedes aegypti
Source: PLoS Genet. 2025 Jun 26;21(6):e1011757. doi: 10.1371/journal.pgen.1011757 (PMC12221180; doi:10.1371/journal.pgen.1011757)
Supplement: S1 Text — (PPTX) [file pgen.1011757.s010.pptx]

## Slide 1
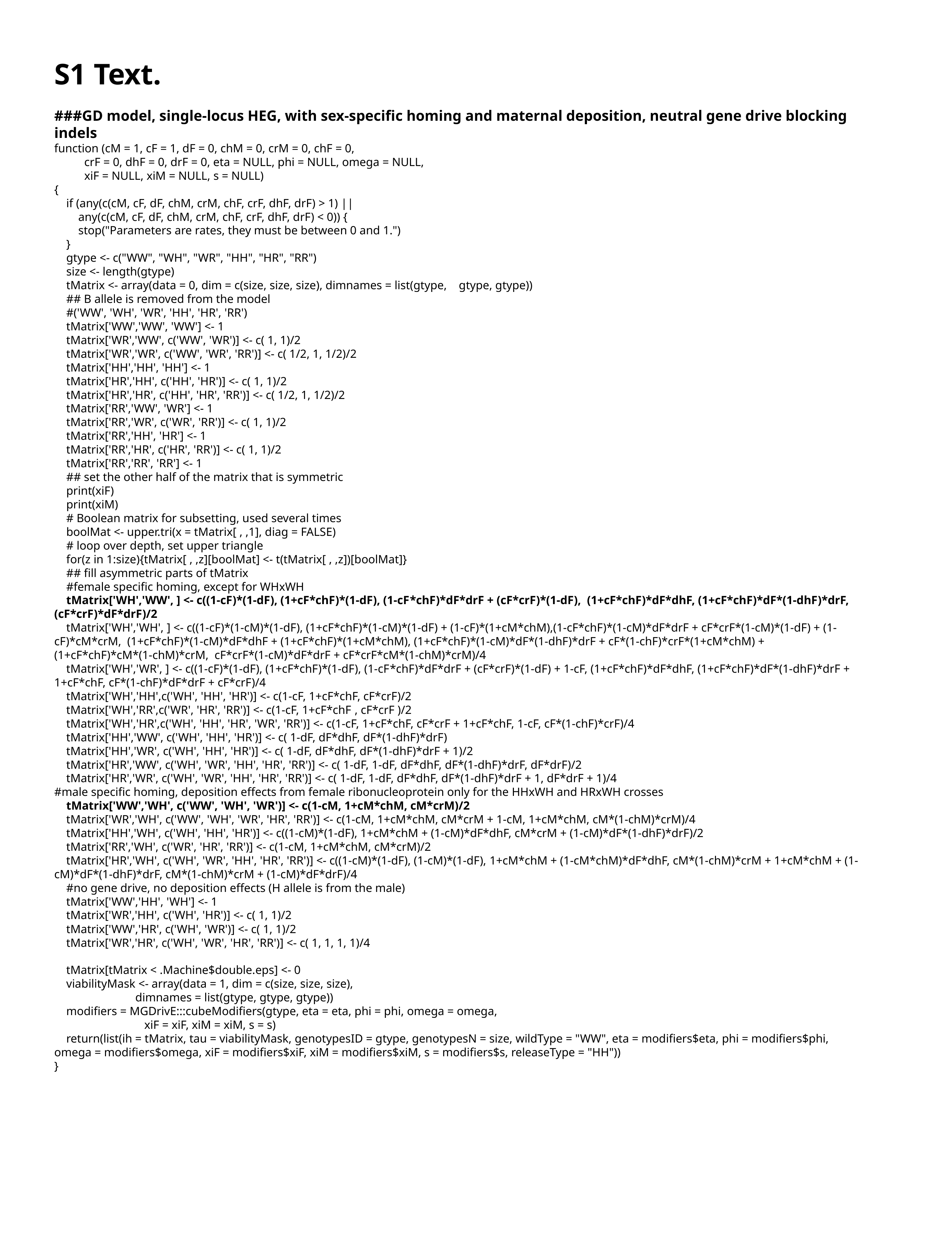

S1 Text.
###GD model, single-locus HEG, with sex-specific homing and maternal deposition, neutral gene drive blocking indels
function (cM = 1, cF = 1, dF = 0, chM = 0, crM = 0, chF = 0,
 crF = 0, dhF = 0, drF = 0, eta = NULL, phi = NULL, omega = NULL,
 xiF = NULL, xiM = NULL, s = NULL)
{
 if (any(c(cM, cF, dF, chM, crM, chF, crF, dhF, drF) > 1) ||
 any(c(cM, cF, dF, chM, crM, chF, crF, dhF, drF) < 0)) {
 stop("Parameters are rates, they must be between 0 and 1.")
 }
 gtype <- c("WW", "WH", "WR", "HH", "HR", "RR")
 size <- length(gtype)
 tMatrix <- array(data = 0, dim = c(size, size, size), dimnames = list(gtype, gtype, gtype))
 ## B allele is removed from the model
 #('WW', 'WH', 'WR', 'HH', 'HR', 'RR')
 tMatrix['WW','WW', 'WW'] <- 1
 tMatrix['WR','WW', c('WW', 'WR')] <- c( 1, 1)/2
 tMatrix['WR','WR', c('WW', 'WR', 'RR')] <- c( 1/2, 1, 1/2)/2
 tMatrix['HH','HH', 'HH'] <- 1
 tMatrix['HR','HH', c('HH', 'HR')] <- c( 1, 1)/2
 tMatrix['HR','HR', c('HH', 'HR', 'RR')] <- c( 1/2, 1, 1/2)/2
 tMatrix['RR','WW', 'WR'] <- 1
 tMatrix['RR','WR', c('WR', 'RR')] <- c( 1, 1)/2
 tMatrix['RR','HH', 'HR'] <- 1
 tMatrix['RR','HR', c('HR', 'RR')] <- c( 1, 1)/2
 tMatrix['RR','RR', 'RR'] <- 1
 ## set the other half of the matrix that is symmetric
 print(xiF)
 print(xiM)
 # Boolean matrix for subsetting, used several times
 boolMat <- upper.tri(x = tMatrix[ , ,1], diag = FALSE)
 # loop over depth, set upper triangle
 for(z in 1:size){tMatrix[ , ,z][boolMat] <- t(tMatrix[ , ,z])[boolMat]}
 ## fill asymmetric parts of tMatrix
 #female specific homing, except for WHxWH
 tMatrix['WH','WW', ] <- c((1-cF)*(1-dF), (1+cF*chF)*(1-dF), (1-cF*chF)*dF*drF + (cF*crF)*(1-dF), (1+cF*chF)*dF*dhF, (1+cF*chF)*dF*(1-dhF)*drF, (cF*crF)*dF*drF)/2
 tMatrix['WH','WH', ] <- c((1-cF)*(1-cM)*(1-dF), (1+cF*chF)*(1-cM)*(1-dF) + (1-cF)*(1+cM*chM),(1-cF*chF)*(1-cM)*dF*drF + cF*crF*(1-cM)*(1-dF) + (1-cF)*cM*crM, (1+cF*chF)*(1-cM)*dF*dhF + (1+cF*chF)*(1+cM*chM), (1+cF*chF)*(1-cM)*dF*(1-dhF)*drF + cF*(1-chF)*crF*(1+cM*chM) + (1+cF*chF)*cM*(1-chM)*crM, cF*crF*(1-cM)*dF*drF + cF*crF*cM*(1-chM)*crM)/4
 tMatrix['WH','WR', ] <- c((1-cF)*(1-dF), (1+cF*chF)*(1-dF), (1-cF*chF)*dF*drF + (cF*crF)*(1-dF) + 1-cF, (1+cF*chF)*dF*dhF, (1+cF*chF)*dF*(1-dhF)*drF + 1+cF*chF, cF*(1-chF)*dF*drF + cF*crF)/4
 tMatrix['WH','HH',c('WH', 'HH', 'HR')] <- c(1-cF, 1+cF*chF, cF*crF)/2
 tMatrix['WH','RR',c('WR', 'HR', 'RR')] <- c(1-cF, 1+cF*chF , cF*crF )/2
 tMatrix['WH','HR',c('WH', 'HH', 'HR', 'WR', 'RR')] <- c(1-cF, 1+cF*chF, cF*crF + 1+cF*chF, 1-cF, cF*(1-chF)*crF)/4
 tMatrix['HH','WW', c('WH', 'HH', 'HR')] <- c( 1-dF, dF*dhF, dF*(1-dhF)*drF)
 tMatrix['HH','WR', c('WH', 'HH', 'HR')] <- c( 1-dF, dF*dhF, dF*(1-dhF)*drF + 1)/2
 tMatrix['HR','WW', c('WH', 'WR', 'HH', 'HR', 'RR')] <- c( 1-dF, 1-dF, dF*dhF, dF*(1-dhF)*drF, dF*drF)/2
 tMatrix['HR','WR', c('WH', 'WR', 'HH', 'HR', 'RR')] <- c( 1-dF, 1-dF, dF*dhF, dF*(1-dhF)*drF + 1, dF*drF + 1)/4
#male specific homing, deposition effects from female ribonucleoprotein only for the HHxWH and HRxWH crosses
 tMatrix['WW','WH', c('WW', 'WH', 'WR')] <- c(1-cM, 1+cM*chM, cM*crM)/2
 tMatrix['WR','WH', c('WW', 'WH', 'WR', 'HR', 'RR')] <- c(1-cM, 1+cM*chM, cM*crM + 1-cM, 1+cM*chM, cM*(1-chM)*crM)/4
 tMatrix['HH','WH', c('WH', 'HH', 'HR')] <- c((1-cM)*(1-dF), 1+cM*chM + (1-cM)*dF*dhF, cM*crM + (1-cM)*dF*(1-dhF)*drF)/2
 tMatrix['RR','WH', c('WR', 'HR', 'RR')] <- c(1-cM, 1+cM*chM, cM*crM)/2
 tMatrix['HR','WH', c('WH', 'WR', 'HH', 'HR', 'RR')] <- c((1-cM)*(1-dF), (1-cM)*(1-dF), 1+cM*chM + (1-cM*chM)*dF*dhF, cM*(1-chM)*crM + 1+cM*chM + (1-cM)*dF*(1-dhF)*drF, cM*(1-chM)*crM + (1-cM)*dF*drF)/4
 #no gene drive, no deposition effects (H allele is from the male)
 tMatrix['WW','HH', 'WH'] <- 1
 tMatrix['WR','HH', c('WH', 'HR')] <- c( 1, 1)/2
 tMatrix['WW','HR', c('WH', 'WR')] <- c( 1, 1)/2
 tMatrix['WR','HR', c('WH', 'WR', 'HR', 'RR')] <- c( 1, 1, 1, 1)/4
 tMatrix[tMatrix < .Machine$double.eps] <- 0
 viabilityMask <- array(data = 1, dim = c(size, size, size),
 dimnames = list(gtype, gtype, gtype))
 modifiers = MGDrivE:::cubeModifiers(gtype, eta = eta, phi = phi, omega = omega,
 xiF = xiF, xiM = xiM, s = s)
 return(list(ih = tMatrix, tau = viabilityMask, genotypesID = gtype, genotypesN = size, wildType = "WW", eta = modifiers$eta, phi = modifiers$phi, omega = modifiers$omega, xiF = modifiers$xiF, xiM = modifiers$xiM, s = modifiers$s, releaseType = "HH"))
}
