# Supplementary material for: Performance of two low-threshold population replacement gene drives in cage populations of the yellow fever mosquito, Aedes aegypti
Source: PLoS Genet. 2025 Jun 26;21(6):e1011757. doi: 10.1371/journal.pgen.1011757 (PMC12221180; doi:10.1371/journal.pgen.1011757)
Supplement: S2 Text — (PPTX) [file pgen.1011757.s011.pptx]

## Slide 1
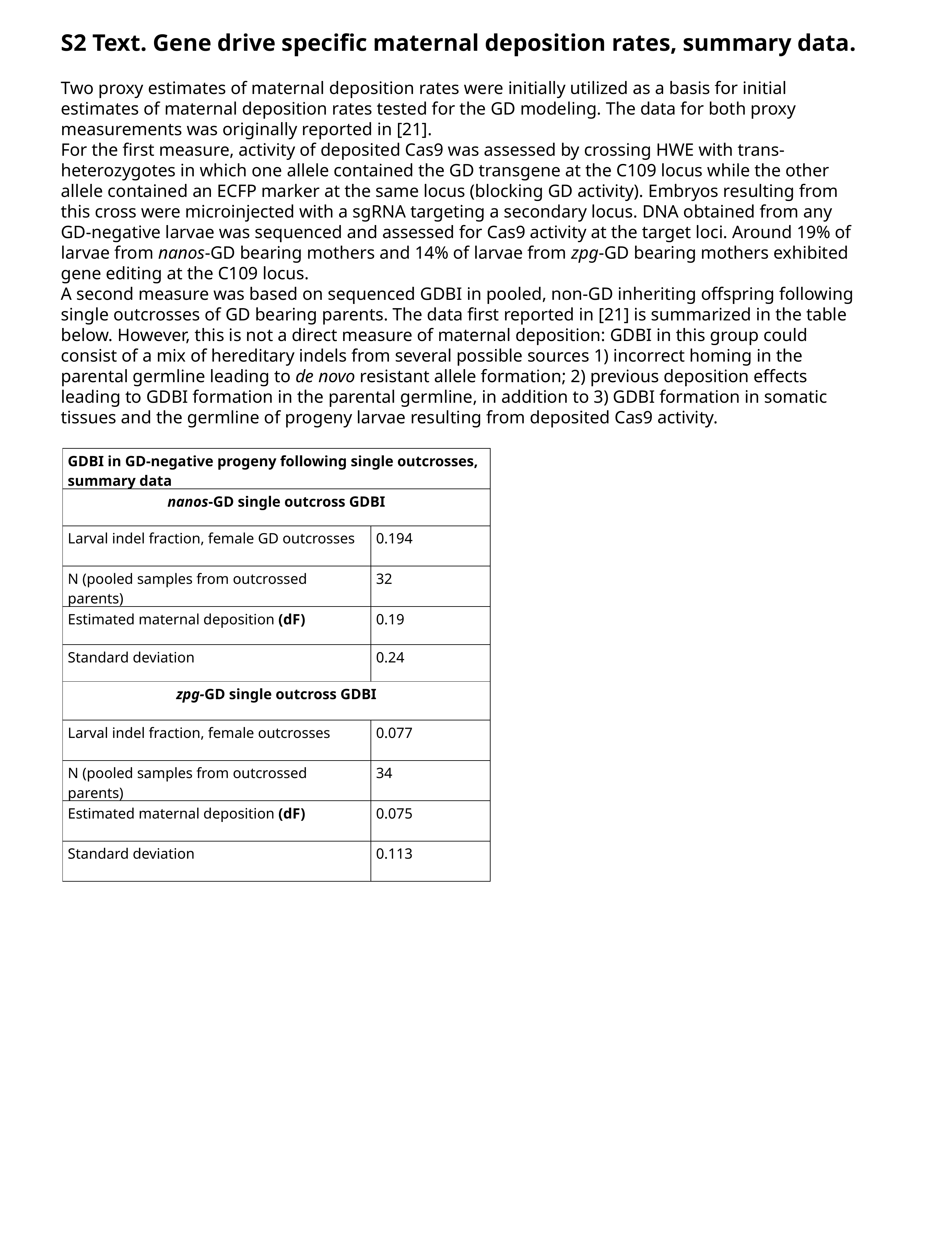

S2 Text. Gene drive specific maternal deposition rates, summary data.
Two proxy estimates of maternal deposition rates were initially utilized as a basis for initial estimates of maternal deposition rates tested for the GD modeling. The data for both proxy measurements was originally reported in [21].
For the first measure, activity of deposited Cas9 was assessed by crossing HWE with trans-heterozygotes in which one allele contained the GD transgene at the C109 locus while the other allele contained an ECFP marker at the same locus (blocking GD activity). Embryos resulting from this cross were microinjected with a sgRNA targeting a secondary locus. DNA obtained from any GD-negative larvae was sequenced and assessed for Cas9 activity at the target loci. Around 19% of larvae from nanos-GD bearing mothers and 14% of larvae from zpg-GD bearing mothers exhibited gene editing at the C109 locus.
A second measure was based on sequenced GDBI in pooled, non-GD inheriting offspring following single outcrosses of GD bearing parents. The data first reported in [21] is summarized in the table below. However, this is not a direct measure of maternal deposition: GDBI in this group could consist of a mix of hereditary indels from several possible sources 1) incorrect homing in the parental germline leading to de novo resistant allele formation; 2) previous deposition effects leading to GDBI formation in the parental germline, in addition to 3) GDBI formation in somatic tissues and the germline of progeny larvae resulting from deposited Cas9 activity.
| GDBI in GD-negative progeny following single outcrosses, summary data | |
| --- | --- |
| nanos-GD single outcross GDBI | |
| Larval indel fraction, female GD outcrosses | 0.194 |
| N (pooled samples from outcrossed parents) | 32 |
| Estimated maternal deposition (dF) | 0.19 |
| Standard deviation | 0.24 |
| zpg-GD single outcross GDBI | |
| Larval indel fraction, female outcrosses | 0.077 |
| N (pooled samples from outcrossed parents) | 34 |
| Estimated maternal deposition (dF) | 0.075 |
| Standard deviation | 0.113 |
